# Identification of novel drought-responsive microRNAs and trans-acting siRNAs from *Sorghum bicolor* (L.) Moench by high-throughput sequencing analysis

**DOI:** 10.3389/fpls.2015.00506

**Published:** 2015-07-09

**Authors:** Amit Katiyar, Shuchi Smita, Senthilkumar K. Muthusamy, Viswanathan Chinnusamy, Dev M. Pandey, Kailash C. Bansal

**Affiliations:** ^1^Indian Council of Agricultural Research-National Bureau of Plant Genetic ResourcesNew Delhi, India; ^2^Department of Biotechnology, Birla Institute of Technology, MesraRanchi, India; ^3^Indian Council of Agricultural Research-National Research Centre on Plant BiotechnologyNew Delhi, India; ^4^Division of Plant Physiology, Indian Council of Agricultural Research-Indian Agricultural Research InstituteNew Delhi, India

**Keywords:** sorghum, microRNAs, tasiRNA, drought, next-generation sequencing, transcriptome

## Abstract

Small non-coding RNAs (sRNAs) namely microRNAs (miRNAs) and trans-acting small interfering RNAs (tasi-RNAs) play a crucial role in post-transcriptional regulation of gene expression and thus the control plant development and stress responses. In order to identify drought-responsive miRNAs and tasi-RNAs in sorghum, we constructed small RNA libraries from a drought tolerant (M35-1) and susceptible (C43) sorghum genotypes grown under control and drought stress conditions, and sequenced by Illumina Genome Analyzer IIx. Ninety seven conserved and 526 novel miRNAs representing 472 unique miRNA families were identified from sorghum. Ninety-six unique miRNAs were found to be regulated by drought stress, of which 32 were up- and 49 were down-regulated (fold change ≥ 2 or ≤ −2) at least in one genotype, while the remaining 15 miRNAs showed contrasting drought-regulated expression pattern between genotypes. A maximum of 17 and 18 miRNAs was differentially regulated under drought stress condition in the sensitive and tolerant genotypes, respectively. These results suggest that genotype dependent stress responsive regulation of miRNAs may contribute, at least in part, to the differential drought tolerance of sorghum genotypes. We also identified two miR390-directed *TAS3* gene homologs and the auxin response factors as tasi-RNA targets. We predicted more than 1300 unique target genes for the novel and conserved miRNAs. These target genes were predicted to be involved in different cellular, metabolic, response to stimulus, biological regulation, and developmental processes. Genome-wide identification of stress-responsive miRNAs, tasi-RNAs and their targets identified in this study will be useful in unraveling the molecular mechanisms underlying drought stress responses and genetic improvement of biomass production and stress tolerance in sorghum.

## Introduction

Sorghum (*Sorghum bicolor* (L.) Moench) is the 4th most important nutritious cereal crop of the world after wheat, rice and maize. Sorghum is widely grown for food, feed, fiber and fuel and serve as a major staple food crop of millions of people, especially in semi-arid tropics including Africa, China, India, Mexico and USA. Drought is one of the major agronomic problems contributing to severe yield losses in sorghum worldwide. Further significant reduction in average yield has been reported due to the drought condition at flowering and grain filling phases, ultimately leading to a reduction in the grains number and size in sorghum. However, few genotypes of sorghum are relatively drought tolerant and known for its adaptation to water-limited environment (Ludlow and Muchow, 1990). Therefore, sorghum may serve as a model crop system for understanding the physiological and molecular mechanisms underlying drought tolerance. Dwindling fresh water resources and projected increase in incidences of drought under global climate change scenario warrant development of climate resilient sorghum varieties and hybrids. This necessitates a thorough understanding of the mechanisms of the physiological and molecular levels that contribute to drought tolerance. The interplay between drought and changes in gene expression has been studied only a few instances in sorghum (Buchanan et al., 2005). Therefore, molecular analysis of gene expression in sorghum needs further studies.

Several classes of small RNAs (sRNAs) regulate gene expression in plants. These include miRNAs (microRNAs), nat-miRNAs (natural antisense miRNAs), hc-siRNAs (heterochromatic small interfering RNAs), tasi-RNAs (trans-acting small interfering RNAs), nat-siRNAs (natural antisense small interfering RNAs), and ra-siRNAs (repeat-associated small interfering RNAs). These sRNAs are involved in growth and differentiation, genome stability, gene expression and defense in plants. miRNAs are coded by *MIR* (*MICRORNA*) genes in plants. *MIR* genes are transcribed by RNA Pol II. The 5′methyl capped and 3′ polyadenylated pri-miRNA transcripts forms stem-loop secondary structures, and these processed into mature miRNAs of typically ~21 nt length by DCL1-SE-HYL1 complex. The biogenesis of tasi-RNAs is triggered by the miRNA that targets the *TAS* (tasi-RNA producing locus) gene transcripts (Allen et al., 2005). Based on the similarity of the mature miRNA sequence, the miRNA (MIR) genes coding for identical or nearly identical mature miRNAs are grouped in to same family. For naming the miRNA in the same family usually zero to two mismatches are considered. Different MIR genes code for same family of miRNAs, the loci is named with the same number and a sequential alphabetical suffix (for more details please refer to Meyers et al., 2008).

Nearby 7000 plant miRNAs have been deposited in the miRBase 21, including 427 from Arabidopsis, 713 from rice, 321 from maize, 241 from sorghum and 525 from brachypodium. Compared with the number of miRNAs identified in rice, miRNAs reported in sorghum are very less. This suggests the potential for identification of many more miRNAs. Sorghum crop is highly tolerant to drought and heat stress, and the extension of *MIR* gene family members, including miR169 family, was suggested as one of the possible reasons for adaptation of sorghum to abiotic stresses (Paterson et al., 2009). Recently, small RNA sequencing approaches has been used to identify spatial and temporal expression pattern of miRNAs in sweet sorghum (Calviño et al., 2011; Zhang et al., 2011a). However, no drought stress regulated miRNAs and tasi-RNAs have been identified so far either through experimental or computational approaches in sorghum. To date, only four *TAS* families (TAS1, TAS2, TAS3, and TAS4) targeted by three miRNAs miR173, miR828, and miR390 have been identified in Arabidopsis. For the identification of genotype-specific and drought-responsive miRNAs, we constructed a small RNA library using RNA samples of two genotypes namely M35-1 (drought tolerant/DT) and C43 (drought susceptible/DS) grown under control and drought stress conditions. The miRNAs and tasi-RNAs were identified by sequencing the small RNA libraries using the Solexa deep sequencing technology combined with bioinformatics analysis. The comprehensive profile of miRNAs and tasi-RNAs, and their regulatory cascades in sorghum identified in this study will provide a basic platform for genetic improvement of sorghum.

## Materials and methods

### Plant materials and stress treatment

Sorghum [*Sorghum bicolor* (L.) Moench] seeds of two genotypes i.e., M35-1 (drought tolerant/DT) (Jogeswar et al., 2006) and C43 (drought susceptible/DS) (Mukri et al., 2010) were obtained from the Indian Institute of Millets Research (formerly known as Directorate of Sorghum Research), Hyderabad, India. Plants were grown at 28–32°C day/night temperature with 12/12 h light/dark period in the National Phytotron Facility of Indian Agricultural Research Institute, New Delhi, India. Plants of both genotypes were irrigated alternately with water and Hoagland's solution at 3 days interval. Thirty days after sowing, drought stress was imposed by withholding irrigation to one set of plants (drought stressed) until the leaf relative water content reached to about 60–65% in both the genotypes. Thus the level of stress experienced by plants measured as RWC was similar in both the genotypes. The fully opened uppermost leaves from untreated (control) and treated (drought stressed) seedlings were harvested, frozen in liquid nitrogen and stored at −80°C in the same day for the construction of small RNA libraries.

### Small RNA library construction and sequencing

Total RNA was isolated from the leaves using the TRIzol reagent (Invitrogen, USA), according to the manufacturer's protocol. The RNA quality was examined using gel electrophoresis(28S:18S > 1.5) and Bioanalyzer (Agilent 2100, RIN = 8.0). Small RNA sequencing libraries were prepared using Illumina Truseq small RNA library preparation kit following manufacturer's protocol. Briefly, one microgram of total RNA was ligated with 3′Illumina RNA adapter using Truncated T4RNA Ligase (NEB), followed by 5′ RNA adapter ligation and RT-PCR. The PCR amplicons were separated in a 6% PAGE along with a custom size ladder to select small RNA fraction. The final library was quantified using Agilent Bioanalyser DNA1000 chip and normalized to 10 nM. Seven pM concentration of the small RNA library was used for cluster generation and sequencing analysis (by Sandor Pvt. Ltd., Hyderabad, India) using the Illumina Genome Analyzer IIx according to the manufacturer's protocol (Illumina Inc., USA). The Illumina FASTQ files generated from this study have been submitted to the EMBL-EBI ArrayExpress (https://www.ebi.ac.uk/arrayexpress) with the accession number **E-MTAB-1630**.

### Bioinformatics analysis of sRNA sequences

Adaptor and low-quality sequences were trimmed as suggested by Sunkar et al. (2008). Sunkar et al. (2008) by using the “sequence file pre-processing tool” from UEA sRNA workbench V2.5.0-Plant version (http://srna-tools.cmp.uea.ac.uk/) (Stocks et al., 2012). High quality trimmed sequences (reads with no “N,” no more than 6 bases with quality score <13) with a length of 16–30 nt were further subjected to remove, if mapped with plant t/rRNAs from “Rfam” (excepted miRNAs), Arabidopsis tRNAs from “The Genomic tRNA Database,” and plant t/rRNA sequences from “EMBL release 95” by using the “filter tool” from UEA sRNA workbench V2.5.0-Plant version.

### Prediction of conserved and novel miRNA members in sorghum

The unique reads were submitted to the miRCat pipeline (UEA sRNA workbench V2.5.0-Plant version). The miRCat (miRNA Categorization) was run with the following parameters: The minimum sRNA abundance: 1 read; the minimum sRNA size: 16 nt; the maximum sRNA size: 30 nt, the minimum length of hairpin: 60 nt; the maximum number genome hits: 16. The 100 nt flanking regions of aligned reads were extracted from the genome and folded using RNAfold (http://rna.tbi.univie.ac.at/cgi-bin/RNAfold.cgi). The miRCat trimmed and analyzed the resulting secondary structure to verify the characteristic miRNA as per the plant miRNAs annotation criteria (Meyers et al., 2008) and executed the following additional checks: (1) The miRNA and miRNA^*^ are derived from opposite stem-arms and form a duplex with two nucleotide 3′ overhangs. (2) The number of mismatches between miRNA and miRNA^*^ should be less than or equal to four. (3) The frequency of asymmetric bulges is one or less and size of the bulge is no more than 2 nucleotides within the miRNA/ miRNA^*^ duplex. The maximum number of occurrences (reads) for a particular miRNA family was denoted as miRNA abundance. The folding structure of precursors was examined using RNA folding/annotation tool (UEA sRNA workbench V2.5.0-Plant version) that uses the Vienna Package to obtain the secondary structure of a precursor sequence and highlighting up miRNA/miRNA^*^ sequences on hairpin structure.

### Differential expression analysis of miRNAs

To compare abundance of miRNAs in control and treatment library, the count of each miRNA was normalized to transcripts per million (TPM) following appropriate statistical method (Audic and Claverie, 1997). (i) Normalization criterion: TPM = (actual miRNA count/total count of clean reads)^*^ 1,000,000. Afterwards, the fold-change and *P*-value of the normalized expression were calculated by using the following formula, (ii) Fold-change criterion: Fold change = log_2_ (miRNA TPM in the treatment library/miRNA TPM in the control library), (iii) *P*-value: The *P*-value of precursor miRNA candidate was tested using randfold (using a cutoff of 0.1) tool (Bonnet et al., 2004) and integrated on the UEA sRNA workbench. The Poisson distribution model was used for estimating the statistical significance of miRNA expression changes under control and treatment conditions. Upregulation of any miRNA expression levels was considered a positive value, while negative values indicate down-regulation. To identify drought-responsive miRNAs, several standards (Eldem et al., 2012) were followed as: (1) normalized count was at least 1 TPM in either control or stress library; (2) *P*-value = 0.01 as the threshold; (3) log_2_ ratio of the normalized count under stress or control libraries was >1 or < −1. Unique (active form of the miRNA) or identical mature miRNAs, generated from two or more homologous miRNA genes were only considered for differential expression analysis.

### Tasi-RNA analysis

To detect phased small RNA clusters corresponding to tasi-RNAs, the “The UEA Small RNA Workbench V2.5.0” (http://srna-workbench.cmp.uea.ac.uk/tools/ta-si-prediction/) (Stocks et al., 2012) was used. Small RNAs (sRNAs) that are not identical to the genome were rejected. A minimum abundance 2 and *p*-value threshold of 0.0001 was used to detect statistically significant clusters. Potential phase-initiators for these *TASs* were predicted by using psRobot program (http://omicslab.genetics.ac.cn/psRobot/) (Wu et al., 2012) and psRNATarget program (http://plantgrn.noble.org/psRNATarget/) (Dai and Zhao, 2011) with default parameters and an assumption that the 10th nucleotide position on the sRNA serving as the phase-initiator corresponds to the cleavage start position of its targeted *TAS*. The overlapping TAS on chromosome was merged as a single cluster. The identical tasi-RNAs within TAS gene were removed and retain only unique tasi-RNAs for further analysis.

### Target gene prediction

The potential targets of sorghum miRNAs were predicted using the psRobot; plant small RNA analysis toolbox (http://omicslab.genetics.ac.cn/psRobot/) (Wu et al., 2012) with strict parameters and psRNATarget (http://plantgrn.noble.org/psRNATarget/) (Dai and Zhao, 2011) with default parameters. For psRobot program, we used the following parameters for the target prediction- Penalty score threshold: 3.0; Five prime boundary of essential sequence: 1; Three prime boundary of essential sequence: 31; Maximal number of permitted gaps: 0; Position after which with gaps permitted: 1. For psRNATarget program, we used the following standards for the target prediction- Maximum expectation: 3.0; Length for complementarity scoring (hspsize): 20; Target accessibility (UPE): 25.0; Flanking length around the target site for target accessibility analysis: 17; Range of central mismatch leading to translational inhibition: 9–11 nt. All the mutual and unique targets were accepted and listed in this article. Further, all the targets regulated by sorghum miRNAs identified in this study were subjected to AgriGO toolkit (http://bioinfo.cau.edu.cn/agriGO/) (Du et al., 2010) to investigate gene ontology enrichment. The singular enrichment analysis (SEA) was performed to find enriched GO terms within annotated miRNA targets.

### RNA-seq library construction, sequencing, and data analysis

One microgram of total RNA was used for the preparation of RNA-Seq library using Illumina TruSeq mRNA library preparation kit following the manufacturer's protocol. In short, poly-A RNA was isolated from total RNA and chemically fragmented. First and second strand synthesis were followed by end repair, and adenosines were added to the 3′ ends. Adapters were ligated to the cDNA and 200 ± 25 bp fragments were gel purified and enriched by PCR. The libraries generated were quantitated using an Agilent Bioanalyzer (DNA 1000 kit; Agilent Technologies, Santa Clara, CA) and a 2 × 101 cycle paired end sequencing (sequenced by Sandor Pvt. Ltd., Hyderabad, India) was performed using an Illumina HiScanSQ sequencer (Illumina Inc.). Initially, raw reads were processed by the NGSQC toolkit (http://www.nipgr.res.in/ngsqctoolkit.html) and high quality reads were subjected to *de-novo* assembly using Trinity assembler (Patel and Jain, 2012). Assembled transcripts were quantified by standard pipeline (Trinity→RSEM→R→DESeq), and those transcripts were removed, which has zero FPKM in all four samples (Anders, 2010; Grabherr et al., 2011; Li and Dewey, 2011). These transcripts were further processed by the transdecoder tool to retrieve the full length coding sequence and subsequent annotated by FastAnnotator (http://fastannotator.cgu.edu.tw/) (Chen et al., 2012). Pathway enrichment analysis was performed for the predicted transcripts by KEGG Automatic Annotation Server (KAAS; www.genome.jp/tools/kaas/) for the classification of spatial and temporal governed pathways. The Illumina FASTQ files generated from this study have been submitted to the EMBL-EBI ArrayExpress (https://www.ebi.ac.uk/arrayexpress) with the accession number **E-MTAB-3571**.

## Results

### Analysis of small RNA population in sorghum

To identify drought stress regulated miRNAs from sorghum, four libraries of small RNAs from two genotypes namely M35-1 (drought tolerant or DT) and C43 (drought susceptible or DS) grown under irrigated and drought stress conditions were constructed and sequenced independently. The relative water content (RWC) of drought stressed seedlings were about 65% in both genotypes (Figure S1 in Supplementary Material), indicating similar level of stress imposed to these genotypes. The Solexa (Illumina) sequencing of small RNA libraries from M35-1 and C43 led to the generation of 19,821,595 and 18,890,943 primary reads, respectively, under well irrigated condition, whereas 22,878,203 and 21,740,738 primary reads, respectively, were generated under drought stress conditions. Initially, the raw reads were filtered for possible adaptor contaminations and subsequently mapped to the sorghum genome. For each library, more than 90% reads were mapped to the genome, suggesting slight contamination in library construction and Illumina sequencing. The remaining raw reads (after adaptor removal) were further filtered for poor quality sequences, invalid sequences and sequences smaller than 16 nt and larger than 30 nt and as a result, a total of 9,472,887 clean reads were obtained which represented 975,457 unique reads under irrigated condition. Similarly, a sum of 7,296,968 clean reads with 944,141 unique reads were also determined from small RNA libraries of drought stress treated seedlings (Table S1 in Supplementary Material). The unique reads (after filtering out for t/rRNA matches) from each library that perfectly mapped (with no mismatch) to the sorghum genome represented the small RNA (sRNA) population.

### Identification of conserved and novel miRNAs

**Figure 1:**
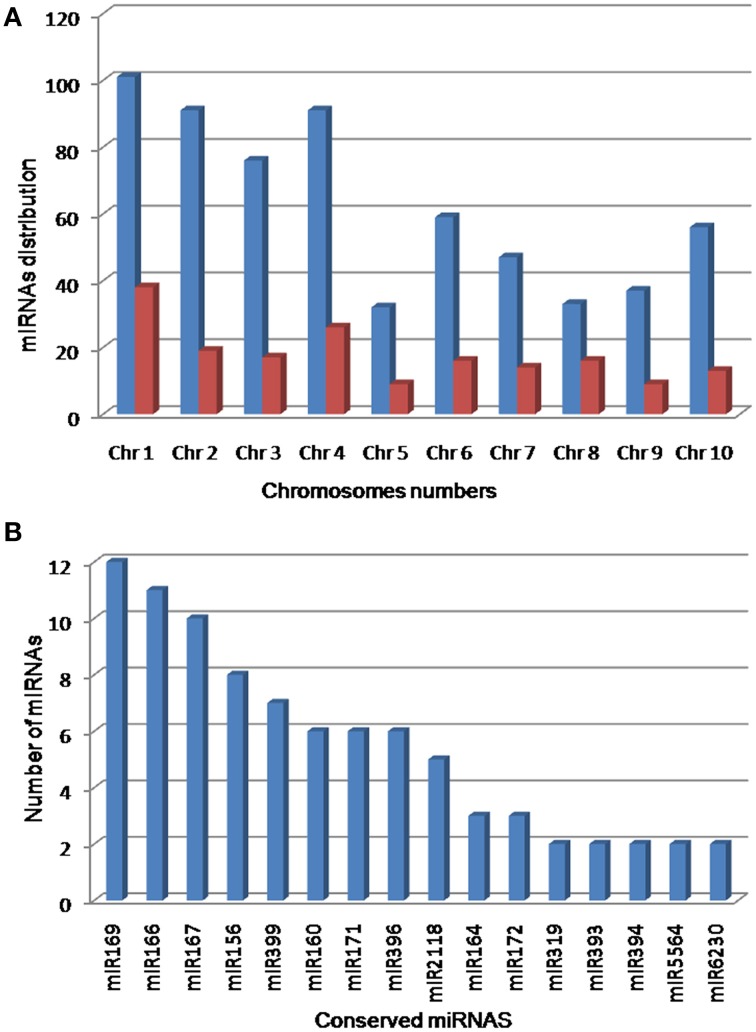
**Distribution of predicted miRNAs**. Chromosomal distribution **(A)** showed that the maximum numbers of miRNAs were predicted from chromosome 1 and 4. Distribution of conserved miRNAs **(B)** showed that miR169, miR166, and miR167 were the most abundant miRNAs in sorghum genome.

To predict miRNAs, all unique reads (gained after filtering) were submitted to the miRCat pipeline (UEA sRNA workbench V2.5.0-Plant version) and were mapped (with no mismatch) against the reference genome of sorghum. The 100 nt flanking regions of aligned reads were extracted from the genome and folded using RNAfold. The resulting secondary structures (potential precursor) were then trimmed and analyzed by miRCat. To recover the putative miRNA candidate, the potential precursors were subjected to a series of stringent criteria suggested for the annotation of plant miRNAs (Ambros et al., 2003; Meyers et al., 2008; Kozomara and Griffiths-Jones, 2013). The probable miRNA candidates that perfectly matched (miRNAs with ≤3 mismatches) with mature miRNAs of sorghum in the miRBase 21 were acknowledged as known miRNAs. The sequences that matched with miRBase entries of other plant species were designated as conserved miRNAs. Finally, the sequences that showed no homology to any previously known and conserved plant miRNAs were denoted as novel miRNA in sorghum. In this study, 97 miRNAs belonging to 26 miRNA families were found identical with the known miRNAs of plant species in miRBase 21. Among these, 32 miRNAs were perfectly matched with mature miRNAs of sorghum and the remaining 65 miRNAs were highly conserved in other plant species and hence are referred to as known and conserved miRNAs, respectively (Table S2 in Supplementary Material). In addition, a total of 526 miRNAs derived from 518 loci identified in this study did not show sufficient homology with any of previously reported plant miRNAs in miRBase 21, and hence classified as novel miRNAs (Table S2 in Supplementary Material). The identification of miRNA^*^ candidates provided further support to consider them as true miRNAs. In this study, 47 and 130 miRNA^*^ were predicted from 97 conserved and 130 novel miRNAs, respectively. A close observation revealed that 541, 499, and 274 reads of miR167h^*^, miR169e^*^, and miR167d^*^ accumulated, respectively. Likewise, 117, 63, and 47 reads of novel miRNAs namely novel-sbi-miR-383^*^, novel-sbi-miR-77b^*^, and novel-sbi-miR-51^*^ accumulated, respectively (Table S2 in Supplementary Material). Furthermore, minimal folding free energy index (MFEI), a sufficient criterion to distinguish miRNA from coding mRNAs and non-coding RNA, i.e., tRNAs and rRNAs, defines that a candidate RNA sequence is more likely to be a miRNA when the MFEI is greater than 0.85 (Zhang et al., 2006). In this study, 96.91% conserved (except for miR166f, miR396f, and miR399b) and 58.56% novel pre-miRNAs of sorghum had an MFEI = 0.85. Additionally, nucleotide bias analysis showed that uracil was the most prominent nucleotide at the beginning in 84 (86.60%) conserved and 177 (33.65%) novel miRNAs. The pre-miRNAs of novel and conserved miRNAs bore a canonical stem-loop structure with free energies ranging from −16 to −131.8 kcal·mol^−1^ (average of −54.29 kcal mol^−1^). The chromosomal distributions of predicted miRNAs are listed in Figure 1.

### Drought-responsive and genotype-specific miRNA

**Figure 2:**
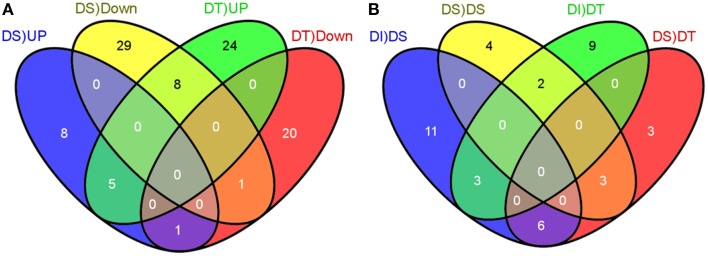
**The Venn diagram illustrates the numbers of common and unique differentially expressed miRNAs induced by drought stress**. Up- and Down-regulated miRNAs (≥2 or ≤ −2 fold changes) under the control and drought stages of drought-susceptible and tolerant genotypes of sorghum are given in **(A)** with the confidence of *P* ≤ 0.05. The numbers of extended drought-responsive miRNAs with the confidence of ≥5 reads at least in one genotype are illustrated in **(B)**.

We compared the normalized count of each miRNAs in a stressed library against the control library to compute the stress regulated expression of miRNAs. The number of mature miRNAs per million clean reads (known as transcripts per million or TPM) was calculated to identify the normalized expression level. A total of 96 unique miRNAs (fold change = 2), belongs to 8 known and 88 novel families, showed differential expression under drought stress as compared to the control, in at least one genotype (Figure 2; Table 1; Table S3 in Supplementary Material). Out of the 96 drought stress regulated miRNAs, 23 and 9 miRNAs were up- and down-regulated, respectively, under the drought stress, in both genotypes. Forty four miRNAs were upregulated in drought tolerant M35-1, while these miRNAs were downregulated in drought sensitive C43 genotype under drought stress. In contrast, 19 miRNAs were downregulated in drought tolerant M35-1, while these miRNAs were upregulated in drought sensitive C43 genotype under drought stress. The novel-sbi-miR-259 was undetectable in M35-1, while it was downregulated in C43 (Figure 2; Table 1; Table S3 in Supplementary Material).

**Table 1:** **Drought stress-responsive miRNAs (fold change >2 “at-lest” in one genotype) and their putative targets are listed**.

**S.No**.	**miRBase ID**	**M (DT)**	**C (DS)**	**Targets**
**UP-REGULATED UNDER DROUGHT IN BOTH M35-1 AND C43**				
1	novel-sbi-miR-44	▲	▲	Acyl-CoA N-acyltransferases (NAT) superfamily protein (Sb02g008700); Dicarboxylate diiron protein, putative (Crd1) (Sb03g011270); Glycosyl hydrolase 9B10 (Sb09g002490)
2	novel-sbi-miR-94	▲	▲	ATP/GTP-binding protein family (Sb10g022230); RING/FYVE/PHD zinc finger superfamily protein (Sb04g029710); fatty acid desaturase 8 (Sb02g043980)
3	novel-sbi-miR-105a-b	▲	▲	ATP binding cassette subfamily B4 (Sb02g019540, Sb03g023740); COBRA-like extracellular glycosyl-phosphatidyl inositol-anchored protein family (Sb02g038740); F-box family protein (Sb04g026045, Sb09g018050); Leucine-rich repeat protein kinase family protein (Sb01g036160); RING-H2 group F2A (Sb05g022230)
4	novel-sbi-miR-108	▲	▲	AUTOPHAGY 6 (Sb03g031280); GDSL-like Lipase/Acylhydrolase superfamily protein (Sb02g040890); methyl esterase 1 (Sb03g045050);NOP56-like pre RNA processing ribonucleoprotein (Sb01g035410, Sb03g044260); peroxisomal ABC transporter 1 (Sb09g000670); zinc finger protein-related (Sb09g027620); SAR DNA binding protein (TC121990); Polyubiquitin containing 7 ubiquitin monomers (CN152471, TC123393, TC127226, TC115840); GDSL-like Lipase/Acylhydrolase family protein (TC117544)
5	novel-sbi-miR-114	▲	▲	DEAD/DEAH box RNA helicase family protein (Sb09g030730); Ethylene-responsive nuclear protein/ethylene-regulated nuclear protein (ERT2) (Sb08g022350); Major facilitator superfamily protein (Sb01g004100); Pentatricopeptide repeat (PPR-like) superfamily protein (Sb06g026460); Stabilizer of iron transporter SufD/Polynucleotidyl transferase (Sb01g011870); Serine/threonine protein phosphatase 3 (Sb10g004490); UDP-D-glucose/UDP-D-galactose 4-epimerase 5 (Sb07g018840)
6	novel-sbi-miR-131	▲	▲	Zinc finger (CCCH-type) family protein/RNA recognition motif (RRM)-containing protein (Sb06g014350); Ubiquitin-specific protease 26 (Sb08g014950); F-box family protein (Sb08g004750); RmlC-like cupins superfamily protein (Sb04g019700); cAMP-regulated phosphoprotein 19-related protein (Sb03g000280)
7	novel-sbi-miR-141	▲	▲	Phosphoinositide 4-kinase gamma 7 (Sb02g027450); zinc transporter 1 precursor (Sb06g028270)
8	novel-sbi-miR-149	▲	▲	Fumarylacetoacetate (FAA) hydrolase family(Sb01g011570); RNA recognition motif (RRM)-containing protein (Sb02g020460); Zinc carboxy peptidase (BM327804); Nucleoside diphosphate kinase (TC134154)
9	novel-sbi-miR-182a-b	▲	▲	Beta glucosidase 13/16 (Sb08g007570, Sb08g007586, Sb08g007650, Sb08g007610); NB-ARC domain-containing disease resistance protein (Sb02g011040); NIMA-related kinase 2 (Sb01g013950); RNA polymerase II, Rpb4, core protein (Sb08g014990); RNA-binding KH domain-containing protein (Sb03g038070); Serine hydroxymethyltransferase 4 (Sb08g019520); Xyloglucan endotransglucosylase/hydrolase 32 (Sb02g033240)
10	novel-sbi-miR-191	▲	▲	RNA polymerase II transcription mediators (Sb10g000410); VQ motif-containing protein (Sb01g005740); SPla/RYanodine receptor (SPRY) domain-containing protein (Sb04g022720); Regulator of Vps4 activity in the MVB pathway protein (Sb03g031550); Ribosomal protein L10 family protein (Sb05g000460); WD-40 repeat family protein/beige-related (Sb04g032555); Transcription factor HBP-1a (bZIP) (TC118669); HAD-superfamily hydrolase (TC123668); RanBPM-like (TC113595)
11	novel-sbi-miR-192	▲	▲	RPM1 interacting protein 13 (Sb01g019820)
12	novel-sbi-miR-205	▲	▲	WRKY DNA-binding protein 21 (Sb01g008550)
13	novel-sbi-miR-221a-h	▲	▲	Polypyrimidine tract-binding protein 2 (Sb03g028150)
14	novel-sbi-miR-224	▲	▲	Chitinase A (Sb02g004650); cytochrome P450, family 96, subfamily A, polypeptide 10 (Sb01g047610); Major facilitator superfamily protein (Sb09g020280); Xylanase inhibitor protein 1 precursor (CD223557, TC116590, TC130018)
15	novel-sbi-miR-229	▲	▲	RNA-binding (RRM/RBD/RNP motifs) family protein (Sb02g034650); auxin response factor 11 (Sb03g000530); Pentatricopeptide repeat (PPR) superfamily protein (Sb06g028710); formate dehydrogenase (Sb10g016920); Golgi-localized GRIP domain-containing protein (Sb02g033040)
16	novel-sbi-miR-255	▲	▲	Lipase-like (TC116103)
17	novel-sbi-miR-263a-c	▲	▲	Hydrolase family protein/HAD-superfamily protein (Sb01g038800); lipase class 3 family protein(Sb04g019260, Sb06g020820); Protein kinase family protein with leucine-rich repeat domain (Sb10g022060)
18	novel-sbi-miR-269	▲	▲	GDSL-like Lipase/Acylhydrolase superfamily protein (Sb03g027670); plastid division2 (Sb02g001890); Gnetum gnemon chloroplast psaC ccsA 4.5S rRNA 5S rRNA 16S rRNA (partial) 23S rRNA and tRNA (AW286089)
19	novel-sbi-miR-313	▲	▲	FtsJ-like methyltransferase family protein (Sb09g028780); NAD(P)-binding Rossmann-fold superfamily protein (Sb01g029980); Glycosyltransferase (TC111346)
20	novel-sbi-miR-322	▲	▲	Phosphoglycerate/bisphosphoglycerate mutase family protein (Sb03g000470); Protein phosphatase 2A, regulatory subunit PR55 (Sb03g027000)
21	novel-sbi-miR-324	▲	▲	Protein kinase family protein with leucine-rich repeat domain (Sb08g005100)
22	novel-sbi-miR-337	▲	▲	SLOW GROWTH 1 (Sb08g008260); Peptidyl-prolyl cis-trans isomerase (TC114053)
23	novel-sbi-miR-383	▲	▲	Alpha/beta-Hydrolases1 superfamily protein(Sb04g002780); BolA-like (EH409271); 1-deoxy-D-xylulose 5-phosphate synthase 1-like (TC116788); squamosa promoter binding protein-like 2/9/14 (Sb05g017510, Sb02g029300, Sb07g026220, Sb03g044160, Sb06g024630, Sb04g004940, Sb10g029190, Sb04g003175, Sb02g028420, Sb07g027740)
**DOWN-REGULATED UNDER DROUGHT IN BOTH M35-1 AND C43**				
1	miR169d-l	▼	▼	AAA-type ATPase family protein (Sb07g002080); nuclear factor Y, subunit A1/A3/A6/A7 (Sb01g011220, Sb01g011220, Sb01g045500, Sb08g021910, Sb01g011220); CCAAT-box transcription factor (TC119556)
2	miR529	▼	▼	Squamosa promoter-binding proteins (Sb07g026220, Sb02g029300); protease-related (Sb09g024450); cellulose-synthase-like C12 (Sb09g025260); development and cell death (DCD) domain protein (Sb02g011270); Ubiquitin fusion protein (TC113579)
3	novel-sbi-miR-57	▼	▼	Heavy metal atpase 5 (Sb04g006600)
4	novel-sbi-miR-111	▼	▼	Pumilio 1 (Sb09g001090); AMP-binding enzyme family protein (CF430224); YT521-B-like family protein (CD209184, TC112887, TC133512)
5	novel-sbi-miR-211	▼	▼	ACT-like protein tyrosine kinase family protein (Sb02g031600); BTB-POZ and MATH domain 2 (Sb10g026770); Cellulose synthase-like D5 (Sb02g036000, Sb02g036010); HAT dimerization domain-containing protein/transposase-related (Sb08g016890); MATE efflux family protein (Sb03g012960); NB-ARC domain-containing disease resistance protein (Sb0019s003010); PGR5-LIKE A (Sb01g000570); protein serine/threonine phosphatases;protein kinases; ATP binding (Sb04g011020); Relative of early flowering 6 (Sb03g043210); Sulfite oxidase (Sb09g003680); UDP-glycosyltransferase 73B4 (Sb09g024630); WRKY DNA-binding protein 49 (Sb03g047350)
6	novel-sbi-miR-245	▼	▼	MATE efflux family protein (Sb03g012960); Pentatricopeptide repeat (PPR) superfamily protein (Sb08g020000); tetratricopeptide repeat (TPR)-containing protein (Sb04g029850)
7	novel-sbi-miR-266	▼	▼	GRAS family transcription factor (Sb01g015165); Protein kinase superfamily protein (Sb04g037460); RING/FYVE/PHD zinc finger superfamily protein (Sb09g003580)
8	novel-sbi-miR-339	▼	▼	Formin homology 1 (Sb02g030000); Cytochrome P450, family 71, subfamily B, polypeptide 37 (Sb08g003380, Sb08g003390, Sb01g041085, Sb03g045960); BTB-POZ and MATH domain 4 (Sb01g005590, Sb02g003880); RING/FYVE/PHD zinc finger superfamily protein (Sb08g003900, Sb04g008370); Highly ABA-induced PP2C gene 2 (Sb01g039890)
9	novel-sbi-miR-387	▼	▼	Aluminium activated malate transporter family protein (Sb04g032070); GRAS family transcription factor (Sb10g000520; Sb01g040270; Sb01g050333; Sb06g024820, Sb04g032570, Sb04g032590, Sb01g029650); CTD-phosphatase-like protein (TC121585)
**UP-REGULATED IN M35 BUT DOWN-REGULATED IN C43**				
1	miR160a	▲	▼	Auxin-responsive (ARF 10, 16, 17) family proteins (Sb06g033970, Sb04g026610, Sb06g022810, Sb01g019130, Sb10g027790); Malic enzyme (TC114069); Sensor protein (BM325690)
2	miR396b-c	▲	▼	Argonaute family protein (sb02g032990); Myosin heavy chain-related protein (sb07g029120); OSBP(oxysterol binding protein)-related protein 1D (sb08g011100); poly(A) polymerase 1 (sb01g012650); Protein phosphatase 2C family protein (sb08g022065)
3	miR396d-e	▲	▼	Argonaute family protein (Sb02g032990); MuDR family transposase (Sb10g010620); Proline-rich spliceosome-associated (PSP) family protein/zinc knuckle (CCHC-type) family protein (Sb04g023010)
4	miR5385	▲	▼	Alba DNA/RNA-binding protein (Sb01g046110); ARM repeat superfamily protein (Sb09g028200); basic helix-loop-helix (bHLH) DNA-binding superfamily protein (Sb03g028030); HXXXD-type acyl-transferase family protein (Sb01g027380); Long-chain fatty alcohol dehydrogenase family protein (Sb01g019470); DNA internalization-related competence protein (CN125947); SH3 domain binding protein (AW924710); Gibberellin regulated protein (BE355997)
5	novel-sbi-miR-4	▲	▼	Auxin response factor 9 (TC123217)
6	novel-sbi-miR-6	▲	▼	ATP-dependent helicase family protein (Sb10g000310); TTF-type zinc finger protein with HAT dimerization domain (Sb01g020840); VQ motif-containing protein (Sb10g026640)
7	novel-sbi-miR-19	▲	▼	Receptor like protein 7 (Sb03g004950); RNA-binding (RRM/RBD/RNP motifs) family protein (Sb06g024100); D-isomer specific 2-hydroxyacid dehydrogenase family protein (Sb06g000620); cytochrome P450, family 97, subfamily B, polypeptide 3 (Sb04g004850); ABC2 homolog 12 (Sb0067s002260)
8	novel-sbi-miR-26	▲	▼	Alanine dehydrogenase/PNT, N-terminal domain containing protein (CD206106); ABC transporter, ATP-binding/permease protein (CD211686); Heat shock 70 kDa protein (AW679261); NAC domain containing protein 76 (Sb07g000470)
9	novel-sbi-miR-41	▲	▼	Auxin transport protein (Sb07g010440); Eukaryotic aspartyl protease family protein (Sb02g038150); Exonuclease-like protein (TC113816); LigA (TC123718)
10	novel-sbi-miR-46	▲	▼	Aldehyde oxidase 4 (Sb02g003720); AMP-dependent synthetase and ligase family protein (Sb01g046790); D-alanine–D-alanine ligase family (Sb02g002970); Leucine-rich repeat transmembrane protein kinase (Sb04g008350); Rad23 UV excision repair protein family (Sb10g009520); Ribosomal protein L2 family (Sb02g009810); AMP-binding enzyme family protein (TC122720); Smr domain-containing protein-like (CF484339); Methionine aminopeptidase (CN150723); Nucleoside diphosphate kinase (TC134154); Protein kinase domain containing protein (TC114900); Zinc transporter (TC114013)
11	novel-sbi-miR-48	▲	▼	UB-like protease 1A (Sb01g033010); Major facilitator superfamily protein (Sb01g046010); Leucine-rich repeat transmembrane protein kinase (Sb02g033810); Amidase family protein (Sb01g025910); FAR1-related sequence 5 (Sb04g022400)
12	novel-sbi-miR-76a-d	▲	▼	NB-ARC domain-containing disease resistance protein (Sb02g005210)
13	novel-sbi-miR-82	▲	▼	Serine carboxypeptidase-like 26 (Sb05g024460); UDP-Glycosyltransferase superfamily protein (Sb01g002870)
14	novel-sbi-miR-87	▲	▼	Purple acid phosphatase 3 (Sb01g041570); RING/FYVE/PHD zinc finger superfamily protein (Sb02g037120); Transcriptional factor B3 family protein/auxin-responsive factor AUX/IAA-related (Sb09g028450); Ubiquitin-specific protease 4 (Sb06g018520)
15	novel-sbi-miR-119	▲	▼	Acyl-CoA N-acyltransferase with RING/FYVE/PHD-type zinc finger domain (Sb10g000260); MuDR family transposase (Sb08g020220); squamosa promoter binding protein-like 2/9/14 (Sb06g024630, Sb07g027740, Sb02g028420, Sb04g003175, Sb10g029190, Sb04g004940)
16	novel-sbi-miR-138	▲	▼	P-loop containing nucleoside triphosphate hydrolases superfamily protein (Sb01g036270); RNI-like superfamily protein (Sb06g017410); S-adenosyl-L-methionine-dependent methyltransferases superfamily protein (Sb06g025840); Cysteine-rich RLK (RECEPTOR-like protein kinase) (Sb09g024140); Dgd1 suppressor 1 (Sb01g035060)
17	novel-sbi-miR-144	▲	▼	Evolutionarily conserved C-terminal region 2 (Sb01g046550); HXXXD-type acyl-transferase family protein (Sb03g040180); YT521-B-like family protein (TC114776, TC128350, TC112012); RR1 cuticle protein 2 (TC119816); Chlorophyll synthase (TC127744)
18	novel-sbi-miR-151	▲	▼	NOL1/NOP2/sun family protein (Sb07g014990); ARM repeat superfamily protein (Sb10g027680); Cytochrome P450, family 71, subfamily A, polypeptide 25 (Sb10g027350)
19	novel-sbi-miR-164	▲	▼	Alpha-amylase-like 3 (Sb03g032830); F-box/RNI-like superfamily protein (Sb02g002776)
20	novel-sbi-miR-259	ND	▼	Trehalose-phosphatase/synthase 7(Sb03g034640); NB-ARC domain-containing disease resistance protein (Sb05g002510); phloem protein 2-A13 (Sb01g031190); Ribosomal protein L27 family protein (Sb09g025720)
21	novel-sbi-miR-176	▲	▼	Amino acid permease family protein (Sb01g034170); Calcium-binding EF-hand family protein (Sb07g023990); RING-box 1 (Sb04g030370); Transducin/WD40 repeat-like superfamily protein (Sb10g004900); GAMYB-binding protein (TC117630); CRISPR-associated autoregulator DevR family (CF486137)
22	novel-sbi-miR-178a-b	▲	▼	HXXXD-type acyl-transfera se family protein (Sb08g005680); ZPR1 zinc-finger domain protein (Sb01g007380); 2-oxoglutarate (2OG) and Fe(II)-dependent oxygenase superfamily protein (Sb10g005230); Trehalose-6-phosphate phosphatase (Sb10g007770); Mitochondrial editing factor 9 (Sb10g008110); HAT dimerization domain-containing protein/transposase-related (Sb08g018265); PHE ammonia lyase 1 (Sb04g026520); Myosin heavy chain-related (Sb01g031580, Sb06g018840); DEAD/DEAH box helicase, putative (Sb04g028500); Cytochrome P450, family 78, subfamily A, polypeptide 7 (Sb01g022690)
23	novel-sbi-miR-180a-c	▲	▼	Cellulose synthase like E1 (Sb02g027610); retinoblastoma-related 1 (Sb07g025760); F-box associated ubiquitination effector family protein (Sb09g002420)
24	novel-sbi-miR-212	▲	▼	Pollen Ole e 1 allergen and extensin family protein (Sb09g026510); Ubiquitin fusion degradation UFD1 family protein (Sb03g023980)
25	novel-sbi-miR-240	▲	▼	Nucleotide-sugar transporter family protein (Sb02g038010); Glycine/D-amino acid oxidase (TC120362)
26	novel-sbi-miR-285	▲	▼	SU(VAR)3-9 homolog 5 (Sb06g024160); Probable Ufm1-specific protease (TC124720)
27	novel-sbi-miR-287	▲	▼	RAD3-like DNA-binding helicase protein (Sb03g026660)
28	novel-sbi-miR-292	▲	▼	FUS3-complementing gene 2 (Sb04g024880); PIF1 helicase (Sb06g016400)
29	novel-sbi-miR-304	▲	▼	ARM repeat superfamily protein (Sb04g038390); NOL1/NOP2/sun family protein (Sb07g014990); Protein phosphatase 2C family protein (Sb02g021050); Diphenol oxidase laccase (CF071060); Armadillo-like (TC122146)
30	novel-sbi-miR-310a-e	▲	▼	NAD(P)-binding Rossmann-fold superfamily protein (Sb01g029950, Sb01g029960, Sb01g029980); Translation elongation factor EF1A/initiation factor IF2gamma family protein (Sb06g032980)
31	novel-sbi-miR-314a-c	▲	▼	NB-ARC domain-containing disease resistance protein (Sb02g005210); Shaggy-like kinase(TC115703)
32	novel-sbi-miR-316	▲	▼	NB-ARC domain-containing disease resistance protein (Sb02g033350)
33	novel-sbi-miR-335	▲	▼	ABC transporter family protein (Sb03g001690); RNI-like superfamily protein (Sb09g022040)
34	novel-sbi-miR-336	▲	▼	SCAR homolog 2 (Sb01g038070); Heat shock factor A2b (CB929268)
35	novel-sbi-miR-340	▲	▼	Cysteine-rich RLK (RECEPTOR-like protein kinase) 40 (Sb08g014920); peroxisomal 3-ketoacyl-CoA thiolase 3 (Sb01g020150); Pre-rRNA-processing protein ipi1 (CD225395); 1-acyl-sn-glycerol-3-phosphate acyltransferase (CF483158); Eosinophil-associated ribonuclease 10(CF430012); CinA domain protein (TC128307); Protein WIR1B (TC127450)
36	novel-sbi-miR-351	▲	▼	SART-1 family (Sb02g040465)
37	novel-sbi-miR-368	▲	▼	Leucine-rich repeat protein kinase family protein (Sb04g007490)
38	novel-sbi-miR-373	▲	▼	Protein of unknown function (Sb09g020740)
39	novel-sbi-miR-385	▲	▼	Phosphate starvation response 1 (Sb02g010520); MYB-related protein 1 (Sb09g023830)
40	novel-sbi-miR-390	▲	▼	31-kDa RNA binding protein (Sb07g024400)
41	novel-sbi-miR-391	▲	▼	Auxin efflux carrier family protein (Sb10g004430)
42	novel-sbi-miR-392a-c	▲	▼	U-box domain containing protein (CD210713); FAD-linked oxidoreductase (TC125560); Bundle sheath cell specific protein (TC124246); Calmodulin-binding transcription activator protein with CG-1 and Ankyrin domains (Sb03g044220); FAD-binding Berberine family protein (Sb10g021700); plectin-related (Sb01g034550); Ubiquitin fusion degradation UFD1 family protein (Sb03g023980)
43	novel-sbi-miR-412	▲	▼	Auxin transport protein (Sb07g010440); response regulator 9 (Sb02g010680); Amine oxidase family protein (TC118219)
44	novel-sbi-miR-413	▲	▼	NB-ARC domain-containing disease resistance protein (Sb05g008250, Sb05g008270, Sb05g008030); Thioredoxin-like (TC118042)
45	novel-sbi-miR-416	▲	▼	HAMP domain (BI074248); 2 3-bisphosphoglycerate-independent phosphoglycerate mutase (CF771695); ATP-binding cassette sub-family B member (CB928529); F-box and associated interaction domains-containing protein (Sb08g004745); Pentatricopeptide repeat (PPR) superfamily protein (Sb08g000870, Sb08g000890)
**DOWN-REGULATED IN M35 BUT UP-REGULATED IN C43**				
1	miR2118e	▼	▲	Keratin-associated protein (TC123298); 4-amino-4-deoxychorismate synthase (BE598671); splicing factor, arginine/serine-rich 4 (SRp75) isoform 2 (TC133348); Disease resistance protein (CC-NBS-LRR class) family (Sb09g004410); NB-ARC domain-containing disease resistance protein (Sb06g028930)
2	miR2275	▼	▲	Pentatricopeptide repeat (PPR) superfamily protein (Sb02g038430); receptor like protein 7 (Sb08g006800); SEC6 (Sb04g027870); Protein kinase superfamily protein (Sb03g028800); Calcium-dependent lipid-binding (CaLB domain) family protein (Sb02g009740); terpene synthase 21 (Sb05g006470); ferrochelatase 1 (Sb04g000740)
3	novel-sbi-miR-36	▼	▲	DNA polymerase epsilon subunit B2 (sb07g022680); heat shock transcription factor (sb01g005250, sb01g042370); LUC7 related protein (sb01g001640); monogalactosyldiacylglycerol synthase type C (sb07g027910); NHL domain-containing protein (sb03g027320); Pollen Ole e 1 allergen and extensin family protein (sb09g026510); Transducin/WD40 repeat-like superfamily protein (sb04g022100); Sarcoplasmic reticulum protein-like protein (TC130664); VMP4 protein (TC115388)
4	novel-sbi-miR-59a-c	▼	▲	Glucose-methanol-choline (GMC) oxidoreductase family protein (Sb04g031910); Leucine-rich receptor-like protein kinase family protein (Sb04g008470); Tetratricopeptide repeat (TPR)-like superfamily protein (Sb01g037150)
5	novel-sbi-miR-64	▼	▲	Alanyl-tRNA synthetase (Sb08g008230); basic leucine-zipper 44 (Sb02g020760); Protein kinase family protein (Sb09g006270)
6	novel-sbi-miR-120a-b	▼	▲	Enolase 1(Sb02g023480); S15/NS1, RNA-binding protein(Sb04g036260); ubiquitin-specific protease 12 (Sb05g022390); uridine-ribohydrolase 2 (Sb03g009290); Walls Are Thin 1 (Sb10g002840)
7	novel-sbi-miR-200	▼	▲	BTB-POZ and MATH domain 2 (Sb05g024650); cytochrome P450, family 87, subfamily A, polypeptide 6 (Sb01g017160); plastidic pyruvate kinase beta subunit 1 (Sb01g028470); RING/U-box superfamily protein (Sb03g030660)
8	novel-sbi-miR-215	▼	▲	Phosphate transporter 3;2 (Sb02g026490); RHOMBOID-like 1 (Sb05g027720); Ribosomal protein S11-beta (Sb06g028330); TPX2 (targeting protein for Xklp2) protein family (Sb02g020830)
9	novel-sbi-miR-223	▼	▲	Short-chain dehydrogenase/reductase (CF072307)
10	novel-sbi-miR-227a-c	▼	▲	Amidase family protein (Sb05g020350); Pleckstrin homology (PH) domain-containing protein (Sb09g030860); S-locus lectin protein kinase family protein (Sb10g002600); ThiF family protein (Sb03g027840); Protein WIR1B (TC127450); Beta-1,3-glucanase (CD208397); Nuclear pore protein (TC132690); Non-ribosomal peptide synthetase modules and related proteins-like (EH407477); J-domain protein (TC125925)
11	novel-sbi-miR-256	▼	▲	Dinitrogenase iron-molybdenum cofactor biosynthesis (TC121074)
12	novel-sbi-miR-268	▼	▲	IQ-domain 25 (Sb03g034245); Late embryogenesis abundant (LEA) hydroxyproline-rich glycoprotein family (Sb04g009840); like SEX4 1(Sb07g019250); non-specific phospholipase C2 (Sb03g046200); phosphate 1 (Sb04g036730); Remorin family protein (Sb01g049810); Ubiquitin domain-containing protein (Sb09g026390, Sb02g022530); C-terminal region family protein (TC130454)
13	novel-sbi-miR-295	▼	▲	Heat shock protein DnaJ (Sb03g024860); Subtilase family protein (Sb02g025810)
14	novel-sbi-miR-297a-c	▼	▲	Gibberellin 20 oxidase (Sb02g003940)
15	novel-sbi-miR-301	▼	▲	Signal recognition particle, SRP9/SRP14 subunit (Sb06g029010)
16	novel-sbi-miR-344	▼	▲	DEA(D/H)-box RNA helicase family protein (Sb02g029690); Inorganic H pyrophosphatase family protein (Sb04g005710); BTB-POZ & MATH domain (Sb05g024650); DCD (Development and Cell Death) domain protein (Sb10g005050); WPP domain interacting protein (Sb07g028540); methyl esterase11 (Sb02g038650); long-chain base (LCB) kinase1 (Sb01g017100)
17	novel-sbi-miR-359	▼	▲	Methionine S-methyltransferase (Sb09g000490); pleckstrin homology (PH) domain-containing protein (Sb01g028690)
18	novel-sbi-miR-360a-c	▼	▲	SNF2 domain-containing protein/helicase domain-containing protein (Sb01g046180); FAD/NAD(P)-binding oxidoreductase family protein (Sb01g046710); Alpha/beta-Hydrolases superfamily protein (Sb02g007580); Flavodoxin-like quinone reductase 1 (Sb09g024730); Adenine nucleotide alpha hydrolases-like superfamily protein (Sb03g005760); Transcription factor jumonji (jmj) family protein/zinc finger (C5HC2 type) family protein (Sb04g036630); GDP-L-fucose synthase 1 (TC126325)
19	novel-sbi-miR-376	▼	▲	ARF GTPase-activating protein (Sb02g029540); Methylthiotransferase (Sb05g023010); NB-ARC domain-containing disease resistance protein (Sb05g004640); RNA-binding CRS1/YhbY (CRM) domain-containing protein (Sb03g013160); Tetratricopeptide repeat (TPR)-like superfamily protein (Sb03g026520); Transketolase (Sb06g004280); ubiquitin-protein ligase 4 (Sb09g022820)

The up arrow head shows upregulation of miRNA under drought, while down arrow head shows downregulation of miRNA under drought; the cell with “ND” and no arrow indicate that miRNA was not detected neither in control nor in drought.

### Targets of known and novel miRNAs

Recently, 64 target genes for 17 miRNAs (Jiangfeng et al., 2010), 125 target genes for 42 miRNAs (Zhang et al., 2011a) and 72 potential target genes for 31 miRNAs (Katiyar et al., 2012) were reported in sorghum. Here we identified more than 1300 unique potential targets for 49 conserved and 383 novel miRNA families in sorghum (Table S4 in Supplementary Material). For the remaining 96 new miRNAs, no target could be identified, which might due to the stringency of target prediction used in this study. The further BLAST analysis identified miRNA targets homologous to conserved target genes of several plant species. The putative target genes were considered to be a key factor in a wide range of biological processes. Inconsistent with previous reports (Rhoades et al., 2002; Bartel and Bartel, 2003; Song et al., 2011), many of the miRNA target genes predicted in this study encode transcription factors belonging to SPB, Zinc finger, WRKY, WD-40, NAC, MYB, HSFs, GRAS, ARFs, and bHLH families. Several targets for novel miRNAs identified in this study were genes with unknown function. Further functional analysis of miRNA-target gene pair will contribute to our understanding of the role of these miRNAs in sorghum.

### Go enrichment analysis of miRNA target genes

**Figure 3:**
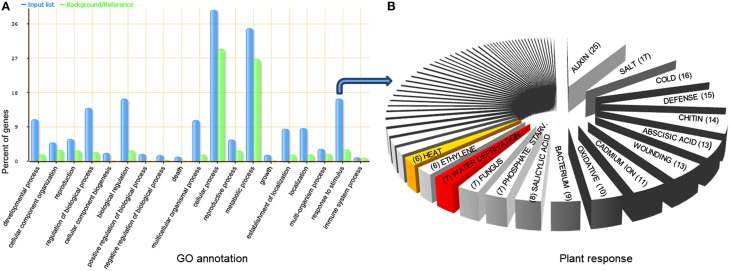
**Gene ontology (GO) analysis of miRNAs target genes identified in sorghum genotypes drought-susceptible and drought-tolerant. (A)** Blue bars indicate the enrichment of miRNA targets in GO terms. Green bars indicate the percentage of total annotated sorghum gene mapping to GO terms. **(B)** Further dissection of “response to stimulus” exposes various stress responsive target genes, including drought (7 genes) and heat (6 genes) highlighted with red and yellow color, respectively.

Widely used standard for functional annotation and enrichment analysis through gene ontology (GO) was carried out for more than 1300 unique potential targets of 472 unique sorghum miRNAs using AgriGO database (Du et al., 2010) with default parameters. Significantly high percentages of these targets were found to be involved in cellular, metabolic, response to stimulus, biological regulation, and developmental processes (Figure 3). Further dissection of “response to stimulus” led to the identification of seven genes with over-represented GO term “response to water deprivation” (GO: 0009414), one with “response to water stimulus” (GO: 0009415), five with “response to heat” (GO: 0009408) and two with “response to heat acclimation” (GO: 0010286) (Figure S3 in Supplementary Material; Table S5 in Supplementary Material). To explore the modulated biological processes between tolerant and sensitive genotypes under drought stress, differentially expressed genes regulated by miRNAs specific to drought tolerant and sensitive genotypes were analyzed separately using GO term enrichment analysis. Gene ontology enrichment analysis was performed using an FDR-adjusted *p*-value of ≤0.05 to select pathways that were statistically enriched with miRNA targets. The significantly enriched GO terms, including response to abiotic stimulus, response to stress, response to stimulus, response to external stimulus, response to organic substances, response to starvation, response to hormone stimulus, response to gravity, response to endogenous stimulus, response to chemical stimulus, and response to auxin stimulus were found among the up- or down-regulated genes in at least one genotype of sorghum (Table S5 in Supplementary Material). Interestingly, three stress-related biological process GO terms varied significantly between tolerant and sensitive genotypes under drought stress. We noticed that GO terms “response to inorganic substance” (GO: 0010035, FDR *p*-value = 0.038) and “response to stress” (GO: 0006950, FDR *p*-value = 0.024) were significantly enriched only in sensitive genotype, whereas “response to abiotic stimulus” (GO: 0009628, FDR *p*-value = 0.045) only in tolerant genotype under drought stress. The results indicate that the inherent preparedness and responsiveness of tolerant cultivar toward drought stress is much higher as compared with sensitive cultivar.

### Digital expression analysis of miRNAs and their targets

**Figure 4:**
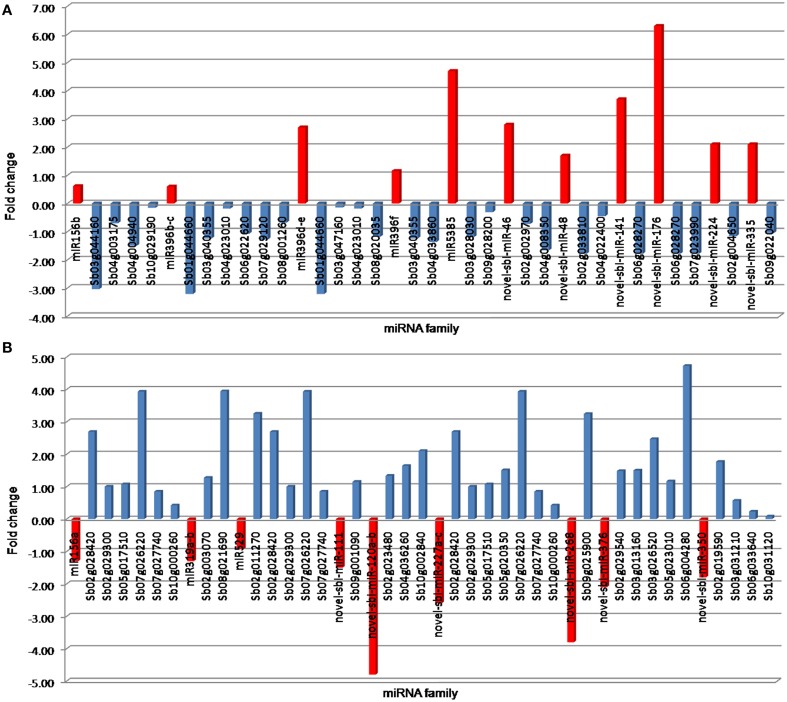
**Negative correlation between drought-responsive miRNAs and their predicted target genes; where (A,B) represents miRNA up, target gene down, and vice versa, respectively**. The miRNA expressions obtained by next-generation sequencing is represented by red color. The color blue represents the expression level of target genes obtained from transcriptome analyses of sorghum.

To investigate the relationship between predicted miRNAs and their targets, expression levels were calculated for randomly selected 20 miRNAs (eight conserved and 12 novels) and their corresponding target genes from small RNA and RNA-Seq sequencing experiments, respectively. A negative correlation (miRNA up, target gene down, and vice versa) was observed between the expression levels of several up- or down-regulated miRNAs and their targets (Figure 4). For instance, the induced expression of miR156b, miR396b-c, miR396d-e, miR396f, miR5385, novel-sbi-miR-46, novel-sbi-miR-48, novel-sbi-miR-141, novel-sbi-miR-176, novel-sbi-miR-224, and a novel-sbi-miR-335 resulted in enhanced level of accumulation of their target genes under drought stress in DT-genotype. Conversely, drought-induced down-regulation of miR156a, miR319a-b, miR529, novel-sbi-miR-111, novel-sbi-miR-120a-b, novel-sbi-miR-227a-c, novel-sbi-miR-268, novel-sbi-miR-376, and novel-sbi-miR-350 led to up-regulation of their target gene. The lists of differentially expressed target genes under drought stress are listed in Table S6 in Supplementary Material.

### Identification of sorghum tass

Phase-initiators direct the cleavage of trans-acting siRNA (tasi-RNA or TAS; a newly identified class of 21 nt short siRNAs) and later initiate the production of tasi-RNA clusters. Initially, we identified 135 unique clusters comprising 155 unique tasi-RNA sequences. The predicted tasi-RNAs were found to be conserved in plants. Further investigation revealed that six phase-initiators (e.g., miR390, miR5567, miR5568, miR6220, miR6225, and miR6230) directing 31 unique *TASs* lead to 64 unique tasi-RNAs (Table S7 in Supplementary Material). The length of the phase-initiator, a key determinant for triggering tasi-RNA biogenesis, is usually 22 nt (except 21 nt long miR390) in Arabidopsis (Chen et al., 2010). A similar exception was observed for sorghum miR390 (21 nt long). The length of other phase-initiators varied from 21 to 24 nt (e.g., 21/24 nt, miR5567; 21 nt, miR5568; 23/24 nt, miR6220, and 23/24 nt, miR6225). The variable length of the phase-initiators suggests the cleavage of double-stranded RNAs (dsRNAs) by multiple Dicer-like (DCL) proteins, thereby generating siRNA classes with different sizes (Axtell, 2009). Biogenesis of tasi-RNAs is dependent on miRNA triggers and requires either dual miRNA target sites (known as “two-hit” model) or single-target site (known as “one-hit” model) in the non-coding RNA precursor (Axtell et al., 2006). While most of the target genes were cleaved by only one miRNA at a single recognition site, we identified two target sites for five *TASs* (e.g., sbiTAS_miR5567/6225a-d and sbiTAS_miR5568/6220a) (Figure S2 in Supplementary Material). The length of each *TAS* in this study is restricted to 251 bp and the corresponding *P*-values varying from 4.47E-08 to 8.20E-05. The targets for tasi-RNAs were predicted by degradome supported psRobot server with default parameters. Predicted target genes for unique 64 tasi-RNAs were found to be involved in plant development and stress responses (Table S8 in Supplementary Material).

## Discussion

### High-throughput sequencing of sorghum small RNAs

**Figure 5:**
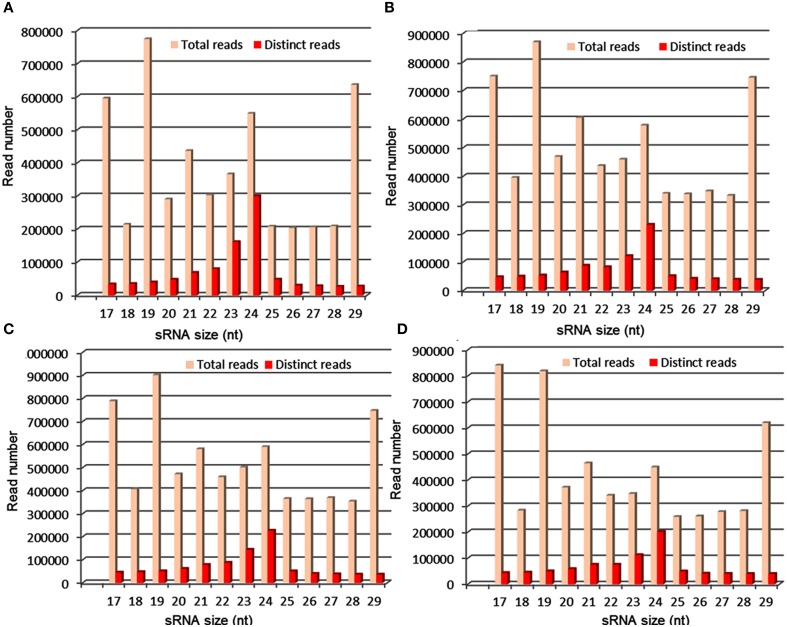
**Distribution of sRNA reads produced from control and drought stress libraries of sorghum; where, (A,B) represents reads distribution in control and stress library of drought-susceptible genotype, respectively. (C,D)** Represent reads distribution in control and stress library of drought-tolerant genotype, respectively. The highest sRNA read abundance was found for 24 nt in all 4 libraries.

In the past few years, high-throughput sequencing is being used to identify miRNAs in several crop plants, including barley, brassica, cowpea, cucumber, maize, peanut, rice, sorghum, soybean, and wheat. In sorghum, 17 (Du et al., 2010), 29 (Zhang et al., 2011a) and 31 (Katiyar et al., 2012) new miRNAs were identified recently. Compared with the number of miRNAs that have been identified in other cereal crops such as rice, only limited miRNAs have been discovered in sorghum. Additionally, drought-regulated miRNAs have not been identified either through experimental or computational methods in sorghum. In the present study, we constructed and sequenced sRNA libraries from seedlings of M35-1 (drought tolerant) and C43 (drought susceptible) genotypes grown under irrigated and drought stress conditions. As a sequencing throughput, the small RNAs population of size 17–29 nt have been found. After eliminating the adapter sequences, the highest read abundance was found for 21–24 nt small RNAs, which is consistent with the size of the DCL enzyme products (Figure 5). The read abundance for each miRNA families highly differed. The miR166, miR167, miR156, and miR399 are the largest miRNA families with 11, 10, 8, and 7 members, respectively, in sorghum. The miR160 and miR396 families have 6 members each (Figure 1; Table S2 in Supplementary Material). Likewise, miR156g^*^ and miR398^*^ were found to accumulate at high levels under drought condition in DS and DT genotypes of sorghum, suggesting that these two miRNA^*^ might function in stress response mechanism irrespective of genotypes. The miR166f^*^, miR167g^*^, miR169e^*^, miR398^*^, and novel-sbi-miR-383^*^ accumulated at high levels exclusively in DS genotype, whereas, miR166g^*^, miR167h^*^, and miR169h^*^ accumulated at high levels exclusively in DT genotype, suggesting the function of these miRNA^*^ in a genotype specific manner. We also observed that the majority of the miRNA transcripts showed higher abundance in tolerant genotype as compared to susceptible genotype under drought treatment (Table S4 in Supplementary Material).

### Monocot- and dicot-abundant miRNAs

Previous studies have shown that some miRNAs are conserved across the plant kingdom, while other miRNAs have been reported in either monocot or dicot plants. In this study, 49 conserved miRNAs belonging to 26 miRNA families were identified from previously deposited miRNAs in miRBase 21 (Table 2). Among these, 30 (61.22%) miRNA families (denoted with the symbol “&” in Table 2) were found to be highly conserved between dicot and monocot plants. The remaining 19 (38.78%) miRNA families (denoted with the symbol “#” in Table 2) were found conserved in monocot plants only. In this study, miR156g-h was identified first time in monocot, which was previously reported only in dicot plant, suggesting the conserved nature of this family in monocot and dicots. Likewise, variants of selected miRNA members, namely miR156b, miR169b, miR396a, and miR399a were found in sorghum as reported previously in monocots. However, other variants of the same family were found to be conserved in dicot plants. This suggests that these miRNAs might have evolved after the divergence of monocots and dicots. In the present study, we found monocot abundant miR156g-h, miR166c-i, miR167f-j, miR169b, miR171a-b, miR172a-c, miR319a-b, miR395, miR396a, miR437, miR529, miR2118a-b, miR2118d, miR2118e, miR2275, miR5385, and miR6221 (denoted with the symbol “@” in Table 2) which were not previously reported in sorghum. Similarly, miRNA families, namely miR437, miR156g-h, miR2118d, miR5385, and miR6221 (denoted with the symbol “$” in Table 2) were identified first time in monocots. In addition, we found that the expression levels of conserved miRNAs were higher than that of unique miRNAs. For example, conserved miRNAs such as miR156c-f, miR156g-h, miR160b-f, miR166c-i, miR166j-k, miR167d-e, miR167f-j, miR396a, and miR398 exhibited high levels of read abundant (more than 750 TPM) in all four libraries. These observations support the earlier conclusion that phylogenetically conserved miRNAs are highly abundant (Sunkar et al., 2008).

**Table 2:** **Conserved^β^ miRNA families in monocot and dicot species**.

**miR family**	**Monocot**										**Dicot**																																									
	**a**	**b**	**f**	**h**	**o**	**s**	**s**	**t**	**t**	**z**	**a**	**a**	**a**	**a**	**a**	**a**	**a**	**a**	**b**	**b**	**b**	**b**	**c**	**c**	**c**	**c**	**c**	**c**	**d**	**g**	**g**	**g**	**g**	**h**	**h**	**h**	**h**	**h**	**l**	**m**	**m**	**m**	**n**	**p**	**p**	**p**	**r**	**s**	**s**	**s**	**v**	**v**
	**t**	**d**	**a**	**v**	**s**	**s**	**b**	**a**	**t**	**m**	**a**	**m**	**t**	**q**	**l**	**t**	**h**	**m**	**n**	**r**	**c**	**g**	**p**	**c**	**r**	**s**	**t**	**m**	**p**	**m**	**s**	**h**	**h**	**a**	**e**	**p**	**t**	**b**	**j**	**d**	**e**	**t**	**t**	**v**	**t**	**p**	**c**	**s**	**l**	**t**	**u**	**v**
	**a**	**i**	**r**	**u**	**a**	**p**	**i**	**e**	**u**	**a**	**u**	**g**	**r**	**c**	**y**	**h**	**y**	**a**	**a**	**a**	**y**	**y**	**a**	**l**	**t**	**i**	**r**	**e**	**r**	**a**	**o**	**b**	**r**	**r**	**x**	**a**	**u**	**r**	**a**	**m**	**s**	**r**	**a**	**u**	**c**	**e**	**o**	**l**	**y**	**u**	**n**	**i**
#miR1432							+			+																																										
&miR156a	+		+		+		+			+						+	+													+						+					+	+					+					
#miR156b							+			+																																										
&miR156c-f	+	+		+		+	+						+					+	+	+			+			+	+	+	+	+				+			+			+	+		+			+		+	+	+	+	
$&miR156g-h							@																							+																						
&miR159							+			+													+																	+					+							
&miR160a		+			+		+										+						+					+													+	+			+		+					+
&miR160b-f	+	+	+		+		+	+	+	+	+		+	+	+	+			+	+			+					+		+										+	+	+	+		+	+	+		+	+	+	+
&miR164a-b	+	+			+		+	+		+			+		+	+			+	+			+			+	+	+		+			+							+	+	+	+		+	+	+	+	+			+
&miR164c	+	+			+		+			+																		+																								
&miR166a-b	+	+			+		+			+															+	+	+																									
&miR166c-i	+	+		+		+	@						+	+	+				+				+			+	+	+	+	+						+		+		+	+		+	+		+		+		+		
&miR166j-k					+		+			+				+														+	+												+	+						+	+	+		
&miR167a-c	+	+				+	+	+		+			+		+		+		+	+			+	+		+	+	+	+	+	+		+						+	+	+	+	+			+	+			+		
&miR167d-e	+	+			+	+	+	+		+			+		+	+							+	+		+	+	+	+	+	+								+		+	+	+		+	+	+					+
&miR167f-j	+	+					@																	+			+			+											+					+						
&miR169a	+				+		+			+				+	+	+			+									+		+										+	+	+	+			+	+		+		+	+
#miR169b							@			+																																										
&miR169c	+	+			+	+	+	+		+																															+											
&miR169d-l	+	+	+		+		+			+			+	+	+	+			+									+				+	+								+		+		+	+		+	+	+		+
&miR171a-b	+						@																																	+										+		
&miR171c-f	+	+	+	+	+		+	+		+				+									+		+			+		+						+	+		+	+	+	+	+		+	+	+		+	+		+
&miR172a-c	+	+			+		@							+	+	+			+	+							+			+										+	+	+	+					+	+	+	+	
#miR2118a-b	+				+		@			+																																										
#miR2118c	+				+		+			+																																										
$#miR2118d							@																																													
#miR2118e	+				+		@			+																																										
#miR2275		+					@																																													
#miR319a-b		+					@																																													
&miR390	+	+			+		+			+			+		+	+			+	+			+			+	+	+		+			+		+				+	+		+	+		+	+	+		+			+
&miR393a-b		+			+		+			+			+		+	+			+				+			+	+	+		+			+				+			+	+	+			+	+	+			+		+
&miR394a-b	+	+			+		+			+			+		+	+	+		+				+			+	+	+		+			+							+	+		+		+	+		+	+	+		+
#miR395	+						@			+																																										
#miR396a	+	+	+		+		@																																													
&miR396b-c	+	+			+	+	+			+	+	+		+	+	+		+	+	+	+		+	+		+		+	+	+				+				+	+	+	+	+	+		+	+		+	+			+
&miR396d-e	+	+			+	+	+			+	+	+		+	+	+					+	+	+			+	+	+	+	+			+					+	+	+	+	+	+		+			+	+			+
&miR396f	+	+			+		+			+			+	+	+	+		+	+	+	+	+		+				+		+										+	+	+	+		+	+	+		+	+		
#miR398	+						+	+		+																																										
#miR399a					+		+			+																																										
&miR399b-d	+	+			+		+			+																+	+			+										+	+				+						+	+
&miR399e					+		+			+																																+					+			+		
&miR399f					+		+			+																																+					+			+		
&miR399g					+		+			+					+	+										+	+			+										+	+	+		+			+				+	+
$#miR437							@																																													
#miR529		+					@			+																																										
$#miR5385							@																																													
#miR5564a-b							+																																													
$#miR6221							@																																													
#miR6230a-b							+																																													

+, Known miRNAs; @, miRNAs reported first time in sorghum; #, Monocot specific miRNAs; &, miRNAs conserved in both monocot and dicot; $, miRNAs reported first time in monocot. ^β^ ata, Aegilops tauschii; bdi, Brachypodium distachyon; far, Festuca arundinacea; hvu, Hordeum vulgare; osa, Oryza sativa; ssp, Saccharum sp.; sbi, Sorghum bicolor; tae, Triticum aestivum; ttu, Triticum turgidum; zma, Zea mays; aau, Acacia auriculiformis; amg, Acacia mangium; atr, Amborella trichopoda; aqc, Aquilegia caerulea; aly, Arabidopsis lyrata; ath, Arabidopsis thaliana; ahy, Arachis hypogaea; ama, Avicennia marina; bna, Brassica napus; bra, Brassica rapa; bcy, Bruguiera cylindrica; bgy, Bruguiera gymnorhiza; cpa, Carica papaya; ccl, Citrus clementina; crt, Citrus reticulata; csi, Citrus sinensis; ctr, Citrus trifoliata; cme, Cucumis melo; dpr, Digitalis purpurea; gma, Glycine max; gso, Glycine soja; ghb, Gossypium herbaceum; ghr, Gossypium hirsutum; har, Helianthus argophyllus; hex, Helianthus exilis; hpa, Helianthus paradoxus; htu, Helianthus tuberosus; hbr, Hevea brasiliensis; lja, Lotus japonicus; mdm, Malus domestica; mes, Manihot esculenta; mtr, Medicago truncatula; nta, Nicotiana tabacum; pvu, Phaseolus vulgaris; ptc, Populus trichocarpa; ppe, Prunus persica; rco, Ricinus communis; ssl, Salvia sclarea; sly, Solanum lycopersicum; stu, Solanum tuberosum; vun, Vigna unguiculata; vvi, Vitis vinifera.

### Response of known and novel miRNAs to drought stress

We compared the small RNA expression profiles of drought tolerant and sensitive genotypes of sorghum and identified 96 drought-responsive miRNAs with more than twofold change at-least in one genotype (Table 1; Table S3 in Supplementary Material). Of the 96 drought regulated miRNAs, 63 miRNAs showed opposite regulation in drought tolerant and drought sensitive genotypes, i.e., 44 of them were upregulated in M35-1 but downregulated in C43, while 19 of them were downregulated in M35-1 but upregulated in C43 (Figure 2; Table 1, Table S3 in Supplementary Material). Among the conserved miRNAs, miR160a, miR169d-l, 396b-c, 396d-e, miR529, miR2118e, miR2275, and miR5385 were found as drought stress responsive in sorghum. The miR160 is known to regulate ARF10/ARF16/ARF17 repressors family and overexpression of miR160 confers auxin hypersensitivity (Turner et al., 2013). In Arabidopsis, ARF17, a target of miR160, negatively regulate the expression of auxin inducible Gretchen Hagen3 (GH3) genes, encoding acyl-acid-amido synthetase which fine-tuning adventitious root initiation in the Arabidopsis (Gutierrez et al., 2012). In cereals, adventitious (nodal/crown) roots, contributes significantly to soil moisture uptake under drought stress (Rostamza et al., 2013). In the present study, we also found that miR160a targets several ARF family members, including ARF10, ARF16, and ARF17. Thus, miR160 upregulation in M35-1 and downregulation in C43, might be contributing to better tolerance of M35-1. We also observed that the downregulation of miR396b-c and miR396d-e in the DS genotype of sorghum as reported previously in rice (Zhou et al., 2010) and cowpea (Barrera-Figueroa et al., 2011), but were upregulated in the DT genotype of sorghum as reported previously in Arabidopsis (Liu et al., 2009) and tobacco (Feng-Xi and Di-Qiu, 2009) under drought-stress. Previous studies showed that miR396a-overexpressing transgenic plants were more drought tolerant than wild-type plants (Liu et al., 2009; Yang et al., 2009). Thus, upregulation of miR396 in DT genotype appears to be contributing to drought stress tolerance of M35-1. Likewise, downregulation of miR529 in both genotypes of sorghum was observed as previously shown in rice (Zhou et al., 2010; Jeong et al., 2011). In addition to the conserved miRNA family members, we found drought regulation of several members of novel miRNA families. These miRNAs might be involved in lineage- or species-specific stress response pathways and functions. In general, many of the miRNA families consist of more than one miRNA gene, which may have identical or diverse mature miRNA sequences. These homologous miRNA genes may functionally diverge from each other during the evolutionary process. For instance, in our study, miR156 and miR164 families showed clear evidence for functional diversification. While one member of miR156 family known as miR156b was induced by drought stress, while another member miR156a was significantly down-regulated in drought sensitive genotype. On the other hand, all members of the miR396 family were up-regulated by drought stress in the tolerant genotype, but not in the sensitive cultivar. Detailed analyses revealed that the majority of the sorghum miRNAs was expressed in a genotype specific manner.

### MicroRNAs regulated targets in response to drought stress

In general, plants respond to environmental stresses by regulating target genes through up- or down-regulation of miRNAs to cope with these stresses. In the present study, 108 targets were predicted for 49 known miRNAs using bioinformatics analysis, and many of these were conserved in plant species, indicating broad conservation of the known miRNA regulatory roles in plants (Table 1; Table S4 in Supplementary Material). In addition to the well-documented conserved targets, a few of the known miRNAs, including miR2118c, miR529, and miR399a were found to target additional genes in sorghum that have not been previously reported. Selected miRNAs, such as miR529 (targeting genes coding for SBP, DCD, cellulase, protease-related, and ubiquitin), and miR398 (targeting genes coding for Cu/Zn SOD, selenium-binding protein, and cytochrome C) showed multiple target genes, indicating that these miRNAs have diverse roles. Furthermore, a single gene may be regulated by several miRNAs. For example, squamosa promoter binding protein (SBP) gene is targeted by miR156a, miR156b, miR156c-f, miR156g-h, miR529, novel-sbi-miR-119, novel-sbi-miR-383, novel-sbi-miR-329, and a novel-sbi-miR-350 in sorghum. In addition, several conserved members of identical miRNA families were found to possess conserved target genes. For instance, all members from the sbi-miR156 and sbi-miR160 family targeted to SBP and auxin response factor (ARF), respectively. Similarly, all members of the sbi-miR399 and sbi-miR164 families targeted phosphate transporter (PHT), and NAC domain containing protein, respectively. Similar results were observed previously in several plant species and these miRNA target genes have been found to be involved in plant growth and/or responses to environmental changes (Unver and Budak, 2009; Xie et al., 2011). The putative targets of the drought regulated miRNAs offered important clues on the drought response in sorghum. For instance, sbi-miR164 (targeted to NAC transcription factor) was found to be downregulated by drought stress at-lest in one genotype of sorghum. This is consistent with previous reports in Arabidopsis and sugarcane (Guo et al., 2005; Raman et al., 2008; Ferreira et al., 2012). NAC transcription factors regulate the development, growth and stress responses, including cold, drought and pathogen attack (Kikuchi et al., 2000; Ooka et al., 2003). Similarly, several droughts regulated miRNAs such as novel-sbi-miR-4, novel-sbi-miR-41, novel-sbi-miR-87, novel-sbi-miR-391, and novel-sbi-miR-412, which target ARFs, were upregulated in DT M35-1 but down regulated in DS C43 sorghum genotype under drought stress. This is consistent with earlier studies that showed stress adaptation by ARFs regulated auxin-mediated mechanisms (Guilfoyle and Hagen, 2007). Proper regulation of auxin transport is critical for drought tolerance (Remy et al., 2013). These results suggest that the predicted targets such as NAC and ARFs play important roles in the drought response in tolerant genotype of sorghum. The suppression of novel-sbi-miR-335 in the sensitive genotype under drought stress could be one of the factors associated with susceptibility of sorghum cultivar C43. The induction of novel- sbi-miR-335 in tolerant genotype under drought stress might contribute to drought-tolerance of the M35-1 cultivar. Moreover, the up-regulation of novel-sbi-miR-389, responsive to dehydration stress in drought tolerant M35-1 cultivar was consistent with earlier observations in barley (Kantar et al., 2010) and soybean (Kulcheski et al., 2011). Some miRNAs such as novel-sbi-miR-360a-c showed up-regulation in drought sensitive C43, but showed down-regulation in drought tolerant M35-1 indicating different adaptive mechanisms by respective genotype. Interestingly, the novel-sbi-miR-266 and a novel-sbi-miR-339 miRNA family showed decreased expression levels under drought stress in both the genotypes. These miRNAs may control a genotype-independent common drought response mechanism. All these miRNA members putatively target a zinc finger protein. Downregulation of novel-sbi-miR-112 (targeted to “basic region/leucine zipper protein-60” or bZIP60) and novel-sbi-miR-254 (targeted to “homeobox-leucine zipper protein-17” or HD-ZIP17) in response to drought may increase the abundance of bZIPs, and contribute to drought tolerance in the DS genotype of sorghum. The importance of bZIPs in stress tolerance of plants was also reported previously (Yang et al., 2009; Golldack et al., 2011). Moreover, miR5385 was up-regulated by drought in the tolerant cultivar, but down-regulated in sensitive cultivar. The predicted target for this miRNA is a basic-helix loop-helix (bHLH) transcription factor. Several reports elucidated the role of bHLH in response to abiotic stresses, e.g., freezing (Chinnusamy et al., 2003), iron deficiency (Long et al., 2010) and salt stress (Li et al., 2010a). Abiotic stresses lead to accumulation of excess concentrations of reactive oxygen species (ROS), resulting in oxidative damage to the cell. Peroxidases help restrict ROS build up and thus oxidative damage to the cells. We predicted peroxidise family as target for six miRNA families, namely novel-sbi-miR-30, novel-sbi-miR-106, novel-sbi-miR-177, novel-sbi-miR-204, novel-sbi-miR-272, and a novel-sbi-miR-217 in sorghum. In tolerant genotype, the expression level of novel-sbi-miR-272 was lower than that of sensitive cultivar during drought stress. The increase of novel-sbi-miR-272 in the sensitive genotype C43 under drought stress may lead to a decrease in the transcript levels of peroxides and thus could be one of the factors associated with vulnerability of this genotype. Several droughts-responsive miRNA families such as novel-sbi-miR-105a-b, novel-sbi-miR-180a-c, and novel-sbi-miR-416 targeted to Kelch repeat-containing F-box protein in sorghum. These proteins are known to be involved in response to biotic and abiotic stresses (Sun et al., 2010; Jia et al., 2012). In sorghum, we observed similar expression patterns for these miRNAs irrespective of genotypes. Three miRNA families (e.g., Novel-sbi-miR-213, miR169c, and a miR169d-l) were down-regulated by drought stress in sensitive genotype C43. These miRNAs targeted to nuclear factor Y (*NFY*) transcription factor. Li et al. (2008) reported that *NFYA5* transcript is strongly induced by drought stress in an abscisic acid (ABA)-dependent manner, whereas miR169 was down-regulated by drought stress through an ABA-dependent pathway. Transgenic plants overexpressing miR169 and *NFYA5* knockout plants exhibited hypersensitivity to drought stress. Thus, *NFYA5* is important for drought tolerance in plants (Li et al., 2008). In contrast, tomato plants over-expressing miR169c exhibited better tolerance to drought due to reduced stomata opening (Zhang et al., 2011b). Thus, drought tolerance may likely involve divergent mechanisms in the different plants. Plants respond to heat or drought stress by the induction of the synthesis of heat-shock proteins (HSPs). HSPs play a crucial role in protecting plants under diverse abiotic stresses (Wang et al., 2004). Four miRNA families (e.g., miR396d-e; novel-sbi-miR-26; novel-sbi-miR-85a-k, and a novel-sbi-miR-336) that targeted to HSPs/HSFs were found to be down-regulated during drought stress in the drought sensitive genotype of sorghum. In contrast, miR396d-e and novel-sbi-miR-336 were found to be up-regulated under drought stress in the drought tolerant genotype of sorghum. In addition, GO analysis exposed the response of several target genes in heat (GO: 0009408), water deprivation (GO: 0009414), water stimulus (GO: 0009415) and heat acclimation (GO: 0010286). The miR156b (targets IRREGULAR XYLEM 1), miR396b-c (targets mitochondrial HSP70-2); novel-sbi-miR-27 (targets HSP21), novel-sbi-miR-55 (targets HOMEBOX 7), novel-sbi-miR-171 (targets early responsive to dehydration 15), novel-sbi-miR-254 (targets nitrate transmembrane transporter), novel-sbi-miR-268 (targets HSP101), novel-sbi-miR-272 (targets HSP1; WRKY; Heat-intolerant 1 and Cytochrome P450), novel-sbi-miR-277 (targets F-box family protein), novel-sbi-miR-329 (targets abscisic acid responsive elements-binding factor 2), and novel-sbi-miR-389 (DEHYDRIN XERO1), were observed as a regulator of drought and heat responsive proteins (Table S5 in Supplementary Material). The novel-sbi-miR-268 and a novel-sbi-miR-272 was found to be up-regulated in sensitive genotype, whereas, down-regulated in the tolerant genotype of sorghum. Likewise, miR396b-c and novel-sbi-miR-254 was found to be down-regulated in sensitive, but up-regulated in the tolerant genotype of sorghum. These results suggested that the two genotypes studied here have differential molecular mechanisms to respond to drought stress. In addition to conserved targets, genotype-specific miRNA regulated gene targets were also observed (Table S4 in Supplementary Material). Although many newly evolved miRNAs that may exhibit weak expression, imperfect processing and lack of targets are believed to serve no biological function, many of them have been shown to target and regulate specific genes or gene families in sorghum (Li et al., 2010b; Pantaleo et al., 2010; Zhang et al., 2010).

### Conserved miR390-TAS3 pathway in sorghum

**Figure 6:**
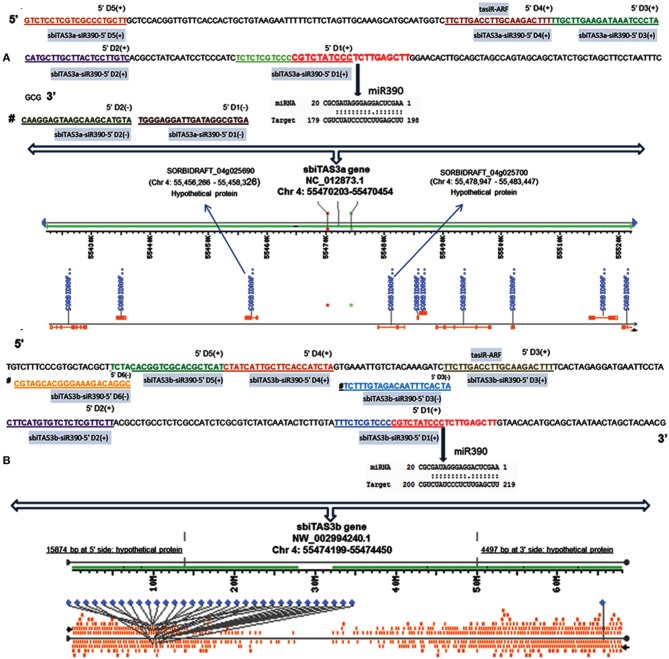
**Diagrammatic representation of miR390 directed *TAS3*a (A) and *TAS3*b (B) genes in sorghum**. Predicted miR30-TAS binding sites are shown in red; all tasi-RNAs are shown in other than red color with an underline; tasi-RNAs have been named by using a standardized nomenclature in which the register is given a number D1, D2… starting at the miRNA target site [17]; the processing direction is noted by a 5′ or 3′ prefix; the orientation is indicated by adding the suffix [+] for the positive (original transcript) strand, or [−] for the negative (RDR generated and denoted by #) strand.

Tasi-RNAs were originally discovered in Arabidopsis, where four *TAS* families (e.g., TAS1/2, TAS3, and TAS4) code for *TAS* transcripts which are targeted by three miRNA families, namely miR173, miR390, and miR828. The tasi-RNA prediction approach includes the detection of phased 21 nt sRNAs, characteristic of tasi-RNA loci and the assessment of statistical significance in term of *P*-value. Using this approach, various research groups have demonstrated the existence of tasi-RNAs in Arabidopsis, rice, grapevine, brassica, apple and soybean. To examine whether there are any homologous genes of predicted *TASs* in sorghum, we aligned 31 *TASs* with non-redundant (nr) nucleotide database. Surprisingly, we did not find homologs of 29 *TASs*. However, two miR390 targeted *TASs* (Table S7 in Supplementary Material) showed significant similarity with *TAS3* gene of *Sorghum bicolor* and *Saccharum officinarum*. Further, these two *TASs* were aligned with Arabidopsis *TAS* genes, and found that sorghum *TAS* is similar to that of *AtTAS3* gene, hence named these as sorghum *TAS3* genes. Thus, the miR390-TAS3 pathway is conserved in sorghum also in addition to the previously reported plants such as Arabidopsis, rice, grapevine, brassica, apple and soybean. Conversely, we did not find miR828-TAS4 and miR173-TAS1/TAS2 pathway in sorghum. In consistency with previous observations that TAS1, TAS2, and TAS4 families do not occur in monocots (Axtell et al., 2006), in our study, we also did not find these tasiRNAs. miR828-TAS4 pathway is widely represented in dicot species (Luo et al., 2012), while the miR173-TAS1/TAS2 pathway has been found to be restricted in Arabidopsis. We mapped *TAS* loci with coding or non-coding regions, and found that 11 *TASs* did not have homology to coding regions, 18 *TASs* showed partial homology to coding regions, and two *TASs* showed significant similarity (>50% query coverage and sequence identity) with coding regions. In addition, miR5567 and miR6225 target *TASs* were found to be highly conserved (Figure S2 in Supplementary Material). We also observed that two putative *TAS3* genes have similar sequences, but different predicted structures. Previous reports disclosed the connection of Arabidopsis tasi-RNAs (TAS1, TAS2, and TAS3) with hypoxia stress (Moldovan et al., 2009). In addition, TAS4-derived siRNAs regulate the biosynthesis of anthocyanins in response to phosphorous deficiency (Hsieh et al., 2009; Luo et al., 2012). Likewise tasi-RNAs from *Pinus taeda* was predicted to target the transcripts of two disease resistance proteins of pine, suggesting its role in the response to pathogens (Lu et al., 2007). However, no drought-stress related tasi-RNAs have been reported in plants. In the present study, we noticed that most of miR390-derived tasi-RNA sequences were present in both DS and DT libraries under control and stress conditions. We assume that neither miR390 nor tasi-RNAs from *TAS3* genes were affected by drought stress. Furthermore, we noticed that all tasi-RNAs derived from miR5567 targeted *TASs*, and miR6230 targeted *TASs* were found only in the control library of drought sensitive genotype. Targets of miRNAs and tasi-RNAs play important roles in many pathways involved in the development or responses to the environment. Our result revealed that 5′ cleavage product of miR390-TAS3 produces two tasi-RNAs [e.g., SbiTAS3a-siR390-5′ D4 (+); sbiTAS3b-siR390-5′ D3 (+)] that target transcripts of auxin response factors (Figure 6). This is consistent with previous reports in Arabidopsis (Allen et al., 2005). Similarly, miR5568-TAS derived tasi-RNA [e.g., SbiTAS-siR5568c-3′ D2 (+)], found in drought-stressed library of drought sensitive genotype, targets to universal stress protein (USP). Genes encoding the universal stress protein domain confer enhanced ability of plants to tolerate stress. The other tasi-RNAs are novel, which will help further broaden the scope of tasi-RNA mediated gene regulation.

## Conclusions

Small RNA-seq in combination with bioinformatic analysis identified 97 conserved and 526 novel miRNAs in sorghum. In addition, we discovered several novels and conserved miRNAs regulated by drought stress. Common as well as genotype-specific miRNA expression patterns were discovered from DS and DT-genotypes of sorghum elucidated the underlying molecular mechanisms and diverse physiological pathways. The targets for predicting miRNAs were found to be involved in cellular, metabolic, response to stimulus, biological regulation, and developmental processes. Additionally, the discovery of two *TAS3* genes (orthologs of Arabidopsis *TAS3* genes) targeted by miR390 revealed conserved miR390-TAS3 pathway in sorghum. The prediction of miR390-TAS3 derived tasiARF suggested their role in the auxin signaling pathway. The outcomes of this study provide valuable information for further functional characterization of miRNAs in response to drought stress in sorghum. Our findings laid the groundwork for functional characterization of drought-responsive miRNAs and manipulating miRNAs or their targets for improving biomass production and stress tolerance in sorghum.

## Author contributions

AK initiated the research, performed the downstream analyses, interpreted the results and drafted the manuscript. SS helped in computational analyses and data management. SM conducted the plant stress treatment and collected the tissues. VC interpreted the results and designed the experiments. DP and KB conceived the idea of the study and drafting the manuscript. All authors have read and approved the manuscript for publication.

### Conflict of interest statement

The authors declare that the research was conducted in the absence of any commercial or financial relationships that could be construed as a potential conflict of interest.

## Abbreviations

AGO: ARGONAUTE
ARF: auxin response factor
DCL: dicer-like protein
DCL1: endoribonuclease Dicer-like 1
DS: drought susceptible
dsRNAs: double-stranded RNAs
DT: drought tolerant
GO: gene ontology
MFEI: minimal folding free energy index
miRNA: microRNA
NGS: next-generation sequencing
PTGS: post-transcriptional gene silencing
RdRP: RNA-dependent RNA polymerase
RISC: RNA-induced silencing complex
RWC: relative water content
siRNA: small interfering RNA
sRNA: small RNA
tasi-RNA: trans-acting siRNAs
TFs: transcription factors
TPM: transcript per million genome-matched reads
UTR: untranslated region.
