# Supplementary material for: Identification of novel drought-responsive microRNAs and trans-acting siRNAs from Sorghum bicolor (L.) Moench by high-throughput sequencing analysis
Source: Front Plant Sci. 2015 Jul 9;6:506. doi: 10.3389/fpls.2015.00506 (PMC4504434; doi:10.3389/fpls.2015.00506)
Supplement: Supplementary file 2 [file FiguresS1toS3.PDF]

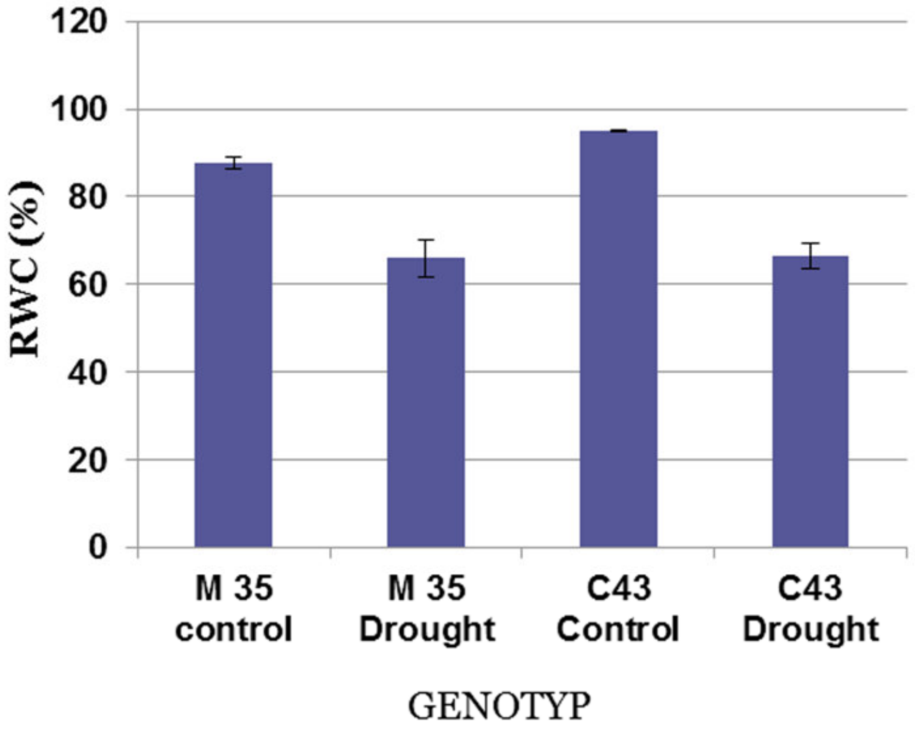

Figure S1 The relative water content of a plant tissue is expressed by RWC (%) = [(FM - DM)/(TM - DM)] * 100, where, FM, DM, and TM are the fresh, dry and turgid masses, respectively, of the tissue.

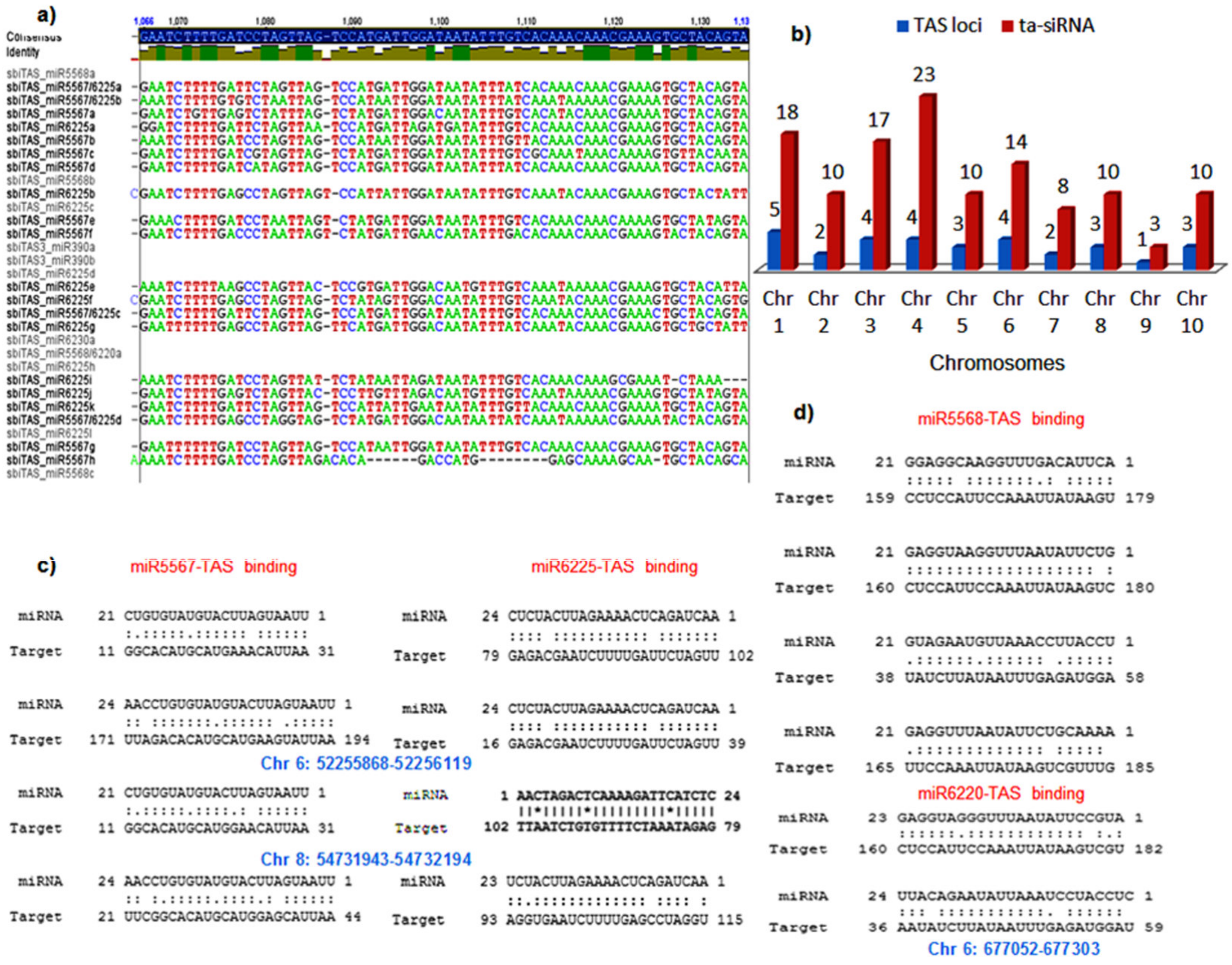

Figure S2 a) sequence alignment of all predicted sorghum TASs. the TASs targeted by miR6225 and miR5567 are shown highly sequence similarity; b) distribution of TASs derived ta-siRNAs on sorghum chromosomes; c-d) graphical representation of binding sites of miR5567/miR6225-TAS and miR5568/miR6220-TAS (known as “two-hit” model); the TASs coordinates highlighted with blue colour.

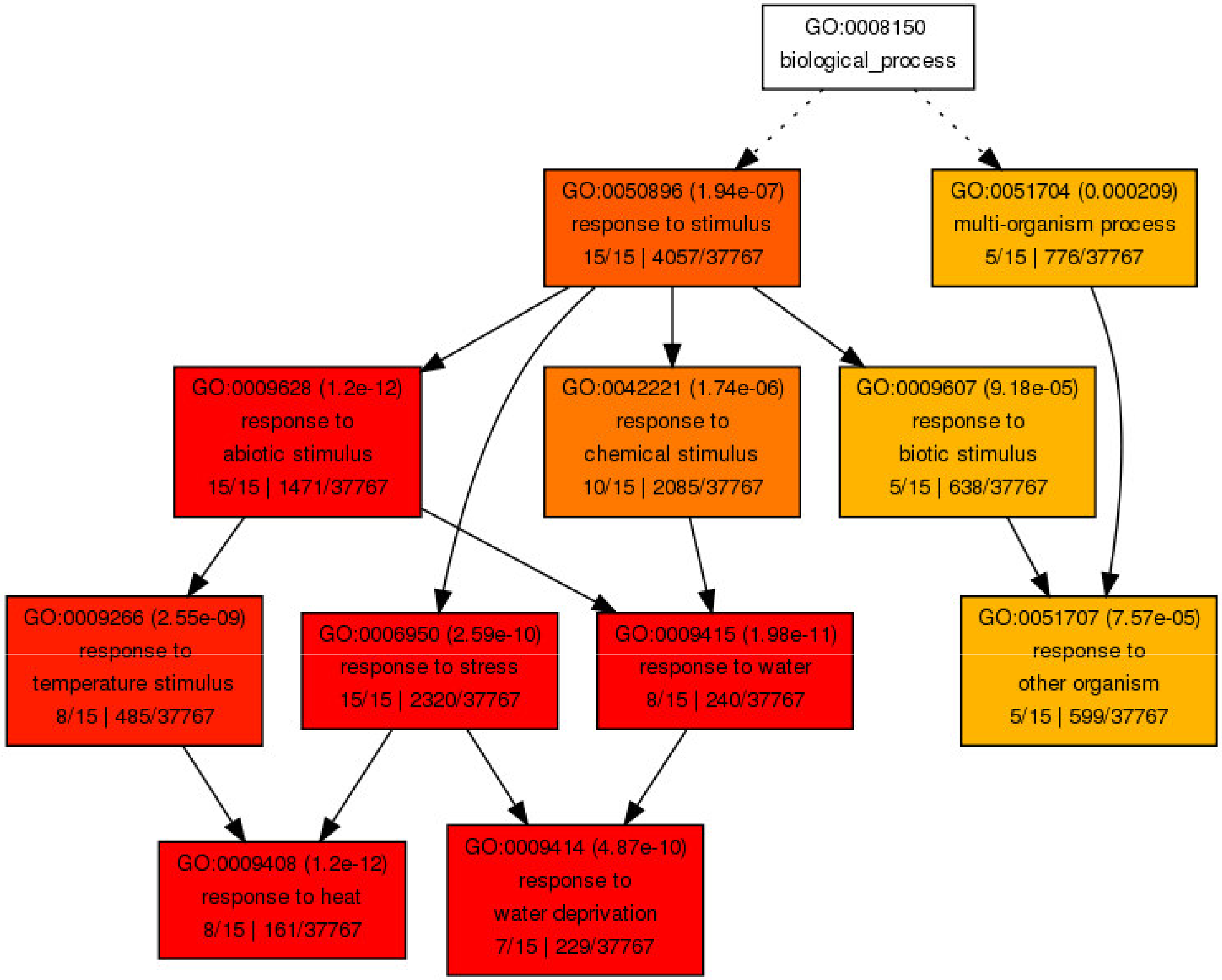

Figure S3 Hierarchical tree graphs of over-represented gene ontology (GO) terms. biological process for target genes in sorghum were constructed using AgriGO. Boxes in the graphs represent GO terms labelled by GO number, term definition and statistical information. Significant terms (adjusted P ≤ 0.05) are coloured. The degree of colour saturation of a box is positively correlated to the enrichment level of the term. Solid, dashed, and dotted lines represent two, one and zero enriched terms at both ends connected by the line.
